# Evaluation of the Chemical Stability, Membrane Permeability and Antiproliferative Activity of Cyclic Diarylheptanoids from European Hornbeam (*Carpinus betulus* L.)

**DOI:** 10.3390/ijms241713489

**Published:** 2023-08-30

**Authors:** Csenge Anna Felegyi-Tóth, Tímea Heilmann, Eszter Buda, Bence Stipsicz, Alexandra Simon, Imre Boldizsár, Szilvia Bősze, Eszter Riethmüller, Ágnes Alberti

**Affiliations:** 1 Department of Pharmacognosy, Semmelweis University, Üllői út 26, 1085 Budapest, Hungary; felegyi_toth.csenge_anna@pharma.semmelweis-univ.hu (C.A.F.-T.); heilmann.timea@stud.semmelweis.hu (T.H.); buda.eszter@stud.semmelweis.hu (E.B.); simon.alexandra@pharma.semmelweis-univ.hu (A.S.); boldizsar.imre@semmelweis.hu (I.B.); riethmuller.eszter@pharma.semmelweis-univ.hu (E.R.); 2 Institute of Biology, Doctoral School of Biology, Eötvös Loránd University, Pázmány Péter sétány 1/C, H-1117 Budapest, Hungary; stipsicz@student.elte.hu; 3 ELKH-ELTE Research Group of Peptide Chemistry, Eötvös Loránd Research Network, Eötvös Loránd University, Pázmány Péter sétány 1/A, H-1117 Budapest, Hungary; szilvia.bosze@ttk.elte.hu; 4 Department of Plant Anatomy, Institute of Biology, Eötvös Loránd University, Pázmány Péter sétány 1/C, 1117 Budapest, Hungary; 5 National Public Health Center, Albert Flórián út 2-6, 1097 Budapest, Hungary

**Keywords:** diarylheptanoid, chemical stability, degradation products, mass spectrometry, PAMPA, antiproliferative activity

## Abstract

Four cyclic diarylheptanoids—carpinontriols A (**1**) and B (**2**), giffonin X (**3**) and 3,12,17-trihydroxytricyclo [12.3.1.1^2,6^]nonadeca-1(18),2(19),3,5,14,16-hexaene-8,11-dione (**4**)—were isolated from *Carpinus betulus* (Betulaceae). Chemical stability of the isolated diarylheptanoids was evaluated as a function of storage temperature (−15, 5, 22 °C) and time (12 and 23 weeks). The effect of the solvent and the pH (1.2, 6.8, 7.4) on the stability of these diarylheptanoids was also investigated. Compounds **2** and **4** showed good stability both in aqueous and methanolic solutions at all investigated temperatures. Only **2** was stable at all three studied biorelevant pH values. Degradation products of **1** and **3** were formed by the elimination of a water molecule from the parent compounds, as confirmed by ultrahigh-performance liquid chromatography–high-resolution tandem mass spectrometry (UHPLC-HR-MS). The permeability of the compounds across biological membranes was evaluated by the parallel artificial membrane permeability assay (PAMPA). Compound **3** possesses a log*Pe* value of −5.92 ± 0.04 in the blood–brain barrier-specific PAMPA-BBB study, indicating that it may be able to cross the blood–brain barrier via passive diffusion. The in vitro antiproliferative activity of the compounds was investigated against five human cancer cell lines, confirming that **1** inhibits cell proliferation in A2058 human metastatic melanoma cells.

## 1. Introduction

**Figure 1:**
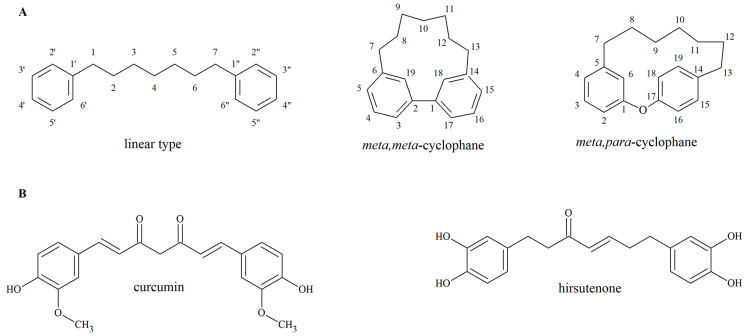
The structures of linear, *meta*,*meta*-cyclophane-type and *meta*,*para*-cyclophane-type diarylheptanoids (**A**); structures of curcumin and hirsutenone (**B**).

Herbs have been used for medicinal purposes since ancient times, and to this day, plants are also potential sources of new drugs. Among plant-derived natural products, diarylheptanoids have gained interest due to their bioactivity, including anticancer [1], neurogenic [2], anti-inflammatory [3], anti-adipogenic [4] and antimicrobial [5] effects. Diarylheptanoids are characterized by a 1,7-diphenylheptane skeleton and can be classified into linear and cyclic forms (Figure 1). The latter can be further divided in two groups: *meta*,*meta*-cyclophanes and *meta*,*para*-cyclophanes, according to the connection of the two phenyl rings [6]. Linear diarylheptanoids are distributed in plants belonging to the families Zingiberaceae and Betulaceae, while cyclic representatives occur in Myricaceae, Aceraceae, Betulaceae and Juglandaceae species [5,6,7]. The number of newly identified compounds is increasing steadily. In their review in 2012, Lv and She summarized more than 400 diarylheptanoids that have been identified in natural sources, among which were 112 cyclic derivatives [7]. In contrast, the paper of Jahng et al. covered nearly 150 cyclic diarylheptanoids [5].

Curcumin (Figure 1B), the yellow pigment of turmeric (*Curcuma longa* L., Zingiberaceae), is one of the most well-known linear diarylheptanoids. Its biological activities have been investigated in numerous in vitro, in vivo and clinical studies [8]; however, its applications are limited because of its poor pharmacokinetic features, high instability and low solubility in aqueous media. Curcumin is degraded quickly through solvolysis and oxidative degradation at ambient temperature, with a half-life of less than an hour, and this process is further promoted by the elevation of the temperature or an alkaline medium [9]. Hirsutenone (Figure 1B), another linear diarylheptanoid aglycone, which is abundant in several species belonging to the Betulaceae family, also lacks chemical stability. The half-life of this compound is less than seven days at room temperature, and it is rapidly hydrolysed in an aqueous solution [10]. Degradation of hirsutenone is further facilitated by the elevation of the temperature: the half-life of hirsutenone in aqueous solution is reduced from 5.78 days at 25 °C to 1.59 days at 50 °C [11].

**Figure 2:**
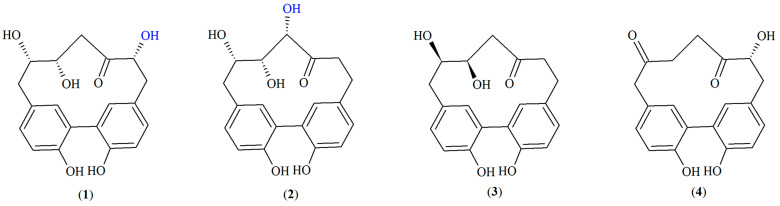
Structures of the investigated cyclic diarylheptanoids: (**1**) carpinontriol A, (**2**) carpinontriol B, (**3**) giffonin X, (**4**) 3,12,17-trihydroxytricyclo [12.3.1.1^2,6^]nonadeca-1(18),2(19),3,5,14,16-hexaene-8,11-dione.

Although there are several stability testing studies regarding linear diarylheptanoids, the chemical stability of the cyclic derivatives is underexplored [12]. Cyclic-type diarylheptanoids are characteristic of species belonging to the genera *Carpinus* [13] or *Corylus* [14,15] in the Betulaceae family. In our previous work, we identified the characteristic *meta*,*meta*-cyclophane-type cyclic diarylheptanoids carpinontriols A (**1**) and B (**2**), giffonin X (**3**) and 3,12,17-trihydroxytricyclo [12.3.1.1^2,6^]nonadeca-1(18),2(19),3,5,14,16-hexaene-8,11-dione (**4**) (Figure 2) in European hornbeam (*Carpinus betulus* L., Betulaceae) for the first time [16].

Although **1**–**4** are known compounds, data on their physical–chemical properties or bioactivities are deficient or completely missing. Lee et al. found that **1** and **2** showed only weak antioxidant activity [13]. In another study*,* **2** inhibited lipid peroxidation induced by H_2_O_2_ in human plasma [17]. In vitro and in vivo antitumor activities of other *meta*,*meta*-cyclophane-type diarylheptanoids isolated from the pericarp of *Juglans nigra* L. [18] or the bark of *Myrica rubra* (Lour) Siebold & Zucc. Ref. [19] have been reported. According to this, the cyclic diarylheptanoids of *C. betulus* may be potential new sources of antitumor agents. Therefore, it is worth exploring their cytotoxic activity and revealing their physical–chemical properties that may restrict their prospective therapeutic use.

Correspondingly, our aim was to determine the aqueous and storage stability of the cyclic diarylheptanoid compounds **1**–**4**. In the storage stability test, effects of the temperature and storage time have been investigated. The influence of the medium, i.e., that of the solvent and the presence of accompanying substances, was also examined. Additionally, the aqueous stability was studied at different physiologically relevant pH values. Our further aim was to determine the ability of the compounds to permeate membranes by passive diffusion, the parallel artificial membrane permeability assays for the gastrointestinal tract (PAMPA-GI) and the blood–brain barrier (PAMPA-BBB) have been used. To further enhance our understanding of the pharmacological properties of cyclic diarylheptanoids, we also aimed to investigate the in vitro antiproliferative activity of the isolated constituents in various human cancer cell lines.

## 2. Results and Discussion

### 2.1. Evaluation of Aqueous Stability at Different pH Values

The stability of the isolated diarylheptanoids (Figure 2) was evaluated in aqueous medium at 37 °C at three biorelevant pH values (pH 1.2 modelling the gastric fluid, pH 6.8 simulating the intestinal fluid, pH 7.4 mimicking the blood and the tissues). Table 1 summarizes the results; compound concentrations are expressed as % values compared to the initial values. To calculate the kinetic parameters [degradation rate constant (*k*) and half-life (*t*_1/2_)], a linear regression model was used, which followed first-order kinetics in line with previous data for diarylheptanoids (Table 2) [11].

Compound **4** was stable only at pH 7.4 after 81 h, while in agreement with our recent results [12], compound **2** remained intact for the whole study at all pH values. Therefore, rate constants and half-lives in these cases have not been determined. At pH 6.8, the amount of compound **4** decreased significantly after 81 h (final concentration 88.9 ± 2.0%), while at pH 1.2, its degradation was more significant both after 9 and 81 h (with final concentrations of 68.5 ± 4.5% and 31.0 ± 7.0%, respectively). Thus, degradation of **4** was remarkably faster at the lower pH values; the half-lives at pH 6.8 and 1.2 differed by one order of magnitude (487.7 h and 54.4 h, respectively). 

The concentration of compound **1** did not show significant changes at pH 1.2 and pH 7.4 after 9 h; however, at the end of the experiment, it displayed significant decomposition (*p* < 0.05; final concentrations were 70.5 ± 2.6% and 71.5 ± 5.2% at pH 1.2 and pH 7.4, respectively). At pH 6.8, compound **1** was not only unstable after 81 h, but already after 9 h (with final concentrations of 75.3 ± 3.0 and 97.4 ± 1.5%, respectively).

At pH 7.4, compound **3** decomposed significantly already after 9 h. On the other hand, its concentration decreased significantly only after 81 h at pH 6.8 and pH 1.2 (with final concentrations of 93.2 ± 2.0% and 83.4 ± 5.3%, respectively). Interestingly, degradation rate constant of **3** was still by one order of magnitude higher at pH 1.2 than at pH 6.8, the compound was the most stable at a pH value of 6.8 (*t*_1/2_ = 826.8 h).

Although compounds **1** and **2** are structural isomers, their stability differs significantly, with **2** staying stable throughout the whole study. The increased stability of compound **2** may be attributed to the electronic stabilization effect of its vicinal triol moiety that may stabilize the compound’s structure. On the other hand, both compounds **1** and **3** comprise a vicinal diol group that may make them prone to undergo pinacol rearrangement [20], especially in an acidic medium. On the contrary, according to the literature data, phenolic compounds are more stable at lower pH values [21]. Nevertheless, the pH of the medium did not significantly influence stability of **1** during our investigation, while for compound **3**, the highest pH value influenced the stability negatively. In the case of component **4**, pH 1.2 differed significantly from the other two pH values; pH 7.4 and pH 6.8 provided better stability. However, no generally prevalent correlation could be determined between the pH values of the medium and the degradation kinetic parameters.

### 2.2. Evaluation of Storage Stability

A further aim of our work was to determine the mid-term (12 weeks) and long-term (23 weeks) stability of the four major diarylheptanoids by evaluating the effects of storage time and temperature. Influence of the medium, i.e., that of the solvent (in aqueous and methanol solutions) as well as that of other accompanying compounds (being present in methanol and ethyl acetate extracts of the hornbeam bark) was also investigated. Aqueous and methanol solutions of the isolated compounds together with hornbeam bark extracts prepared with ethyl acetate and methanol were stored at 22, 5 and −15 °C. The storage temperatures were chosen to represent common storage conditions such storage at ambient temperature, in a refrigerator or in a freezer, respectively.

The methanol and aqueous solutions (SM and SA) of the isolated compounds **2** and **4** did not show significant differences when comparing the initial concentration data with values of weeks 12 and 23 (Table 3). Based on this and the lack of degradation products in their chromatograms, **2** and **4** were considered to be stable. The amount of compound **4** increased when being present in methanol and ethyl acetate extracts (EM and EE) that also comprise further accompanying constituents. This elevation can be explained by the degradation of component **1** that was converted into **4** (see Section 2.3).

In case of the SM and SA solutions of the isolated compounds **1** and **3**, samples showed statistical differences both in the mid- and long-term studies when compared to the initial concentration values. Therefore, the effects of the temperature and the medium on the stability of these compounds were examined; results are shown in Table 3 and Supplementary Figure S1.

After 12 weeks of storage, the concentrations of compound **1** in its methanol and aqueous solutions showed significant differences when stored at 22 °C, as compared to the samples stored at 5 °C. No significant concentration differences were detected for **1** between SA and SM samples stored at temperatures 5 °C and −15 °C. In the case of the methanol extract, the storage temperature did not influence the concentration of compound **1** after 12 weeks.

After 23 weeks of storage, the concentration of **1** decreased significantly in all solutions and extracts at all temperatures, when compared to the initial values. However, lower storage temperatures (both 5 and −15 °C) provided higher stability for the samples. Similarly, when stored for 23 weeks, the concentrations of **3** were statistically lower than the starting concentrations, except for the EE sample stored at 5 °C as well as the SA and EM samples stored at −15 °C.

Moreover, the concentration differences of **1** in the SM and SA solutions were significantly higher at all investigated temperatures than in the ME and EE extracts after 12 weeks of storage. The complex media of the bark extracts provided significantly higher stability in the medium-term at all studied temperatures for **1**. A similar pattern could also be observed at 22 °C after 23 weeks of storage, while both at 5 °C and −15 °C, a concentration decrease of **1** in the aqueous solution was equal to that in the ME and EE extracts.

The matrices of the bark extracts also allowed for appropriate stability for **3** at all storage temperatures after 12 weeks. In the long-term studies (after 23 weeks), the methanol solution of **3** showed significant concentration differences at higher storage temperatures (22 and 5 °C) when compared to the other media (SA, EM and EE). The 23-week storage at 22 °C also intensified the degradation of **3** in the aqueous solution when compared to temperatures of 5 and −15 °C.

Analysing the degradation kinetic parameters of the pure diarylheptanoids **1** and **3**, we can state that the *k* value decreases, and the *t*_1/2_ value increases as the temperature decreases (Table 4). The thermal degradation of **1** and **3** in aqueous and methanolic solutions follows first-order kinetics, in which the degradation rate depends on the temperature. Our results are in agreement with other studies that found that diarylheptanoids are prone to temperature-dependent degradation [10,11].

Comparing the effects of the medium, *k* values of **1** were lower in the aqueous solution than in the methanolic solution (e.g., 4.47 × 10^−3^ vs. 1.40 × 10^−2^ week^−1^ at 5 °C, for SA and SM respectively) (Table 4). Thus, it was concluded that the aqueous medium provided higher stability. This effect was even more pronounced for compound **3**, e.g., calculated half-lives were 1386.29 vs. 97.63 weeks at 5 °C in aqueous and methanolic solution, respectively.

### 2.3. Characterization of the Degradation Products by UHPLC-HR-MS/MS

The structural analysis of the degradation products formed in the storage and pH stability studies was performed by ultrahigh-performance liquid chromatography–high-resolution tandem mass spectrometry (UHPLC-HR-MS/MS) measurements. The chromatographic and mass spectrometric data of the original constituents and the degradation products are presented in Table 5. The high-resolution electrospray ionization mass spectrometry (HR-ESI-MS) and HR-MS/MS spectra of the isolated compounds and their degradation products are shown in the Supplementary Material (Supplementary Figures S2–S13).

**Scheme 1:**
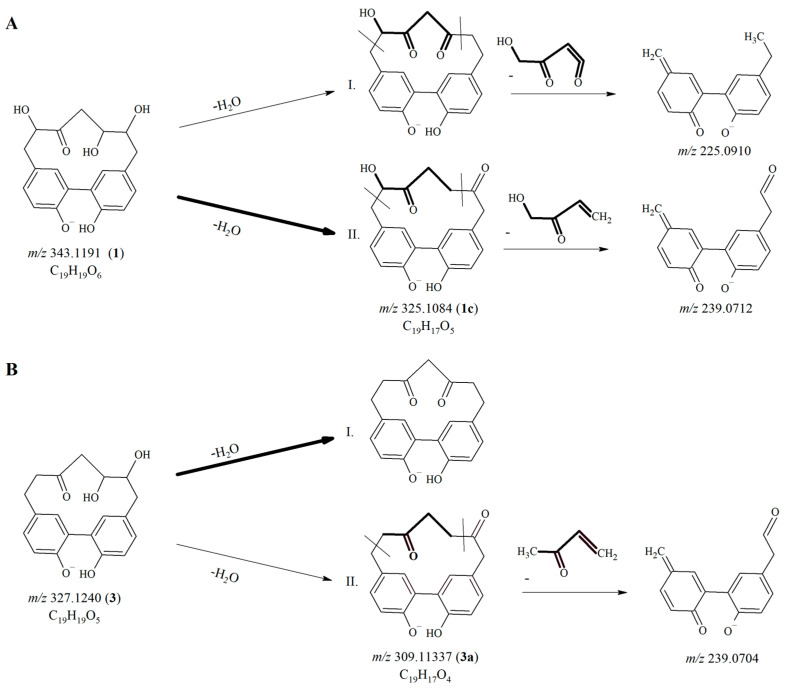
The possible degradation pathways of compound **1** and the proposed mass spectrometric fragmentation of the degradation product **1c** (**A**); the possible degradation pathways of compound **3** and the proposed mass spectrometric fragmentation of the degradation product **3a** (**B**). Degradation product numbers refer to Table 5.

In case of **1** and **3**, new compounds **1c** and **3a** appearing in the chromatograms presented molecular ions bearing *m*/*z* values 18 Da less than the molecular ions of the original compounds. The deprotonated molecular ions of **1c** and **3a** (at *m*/*z* 325.1076 [M − H]^−^ and *m*/*z* 309.1127 [M − H]^−^, respectively), refer to the elimination of a water molecule from their corresponding parent compounds **1** (*m*/*z* 343.1191) and **3** (*m*/*z* 327.1240). In Scheme 1, two possible degradation pathways are depicted for both **1** and **3**, highlighting the characteristic structural differences of the hypothetical products.

As a common structural element, a vicinal diol group is present in the heptane chain of both compounds **1** and **3**, which may be the source of the cleaved water molecule. However, the proposed degradation can undergo through different pathways. The common vicinal diol moiety implies that the pinacol rearrangement is one possible pathway for both **1** and **3**, particularly in an acidic medium [20]. However, when the pH is neutral, there is only a slight chance for the pinacol rearrangement to occur. At the same time, another possible mechanism is for example the radical oxidative degradation [22]. Nevertheless, there is also a possibility that both degradation pathways (or even other mechanisms) may occur at different pH values.

**Table 5:** HR-MS data of the diarylheptanoids **1** and **3** and their degradation products.

No.	[M − H]^−^ (*m*/z) Experimental	[M − H]^−^ (*m*/z) Calculated	Error (ppm)	Molecular Formula	Fragment Ions (*m*/z)
**1**	343.1199	343.1182	3.75	C_19_H_19_O_6_	283.0976 (C_17_H_15_O_4_), 271.0977 (C_16_H_15_O_4_), 269.0820 (C_16_H_13_O_4_), 241.0869 (C_15_H_13_O_3_), 211.0758 (C_14_H_11_O_2_)
**1a**	361.0927	361.0923	2.37	C_18_H_17_O_8_	343.0812 (C_18_H_15_O_7_), 285.0769 (C_16_H_13_O_5_), 258.0534 (C_14_H_10_O_5_),
**1b**	345.0977	345.0974	2.25	C_18_H_17_O_7_	327.0872 (C_18_H_15_O_6_), 309.0764 (C_18_H_13_O_5_), 285.0767 (C_16_H_13_O_5_), 258.0531 (C_14_H_10_O_5_), 225.0549 (C_14_H_9_O_3_)
**1c**	325.1084	325.1076	4.06	C_19_H_17_O_5_	269.0820 (C_16_H_13_O_4_), 253.0862 (C_16_H_13_O_3_), 241.0865 (C_15_H_13_O_3_), 239.0862 (C_15_H_11_O_3_), 225.0910 (C_15_H_13_O_2_), 211.0759 (C_14_H_11_O_2_)
**3**	327.1240	327.1233	4.05	C_19_H_19_O_5_	269.0821 (C_16_H_13_O_4_), 267.1028 (C_17_H_15_O_3_), 253.0866 (C_16_H_13_O_3_), 239.0716 (C_15_H_11_O_3_), 211.0758 (C_14_H_11_O_2_)
**3a**	309.1134	309.1127	4.10	C_19_H_17_O_4_	267.1020 (C_17_H_15_O_3_), 253.0876 (C_16_H_13_O_3_), 225.09131 (C_15_H_13_O_2_), 211.0758 (C_14_H_11_O_2_)

Position of the cleavage of the water molecule could also be proposed, based on the mass spectrometric fragmentation pathway of cyclic diarylheptanoids [16]. The more likely degradation pathways have been highlighted in Scheme 1 by drawing bold arrows. In case of compound **1** (Scheme 1A), the putative degradation product generated through pathway I would present a fragment ion at *m*/*z* 225.0910, while pathway II would result in the formation of a degradation product showing a fragment ion at *m*/*z* 239.0862. Unfortunately, the HR-MS spectrum of the degradation product **1c** presented both fragment ions, though with different intensities. Since the retention time and mass spectrum of compound **1c** corresponds with that of compound **4**, pathway II taking place seems to be more likely. This assumption was further affirmed by the observation that in hornbeam bark extracts the amount of **1** decreased, while that of **4** increased over time during the storage stability assays. 

According to mass spectrometric fragmentation patterns of cyclic diarylheptanoids [16], only pathway II would result in the formation of a degradation product for compound **3,** which could present a characteristic fragment ion at *m*/*z* 239.0704. However, this ion was not detected in the mass spectrum of the actual degradation product **3a**; thus, it was deduced that only pathway I could take place (Scheme 1B).

Although the common structural element of compounds **1** and **3** (i.e., the vicinal diol group) indicated that the same degradation pathway should take place for both **1** and **3**, our results did not confirm this. A possible explanation is the electronic stabilization effect, which may stabilize a compound’s structure or shift the equilibrium toward a degradation product. For example, the stabilizing effect of the vicinal triol moiety may be responsible for the increased stability of compound **2**. Similarly, the additional vicinal carboxylic acid moiety of compound **1** may alter the mechanism of degradation from that of compound **3**.

Two additional degradation products with the molecular formulas of C_18_H_17_O_8_ and C_18_H_17_O_7_ were detected in the chromatogram of **1**, referring to the loss of a carbon-containing moiety and further oxidation mechanisms. In case of compound **4**, degradation products were not detected, despite the significant decrease in the initial concentration (the final concentration of **4** at pH 1.2 was 31.0 ± 7.0%).

### 2.4. Parallel Artificial Membrane Permeability Assay (PAMPA) Studies

The ability of the isolated cyclic diarylheptanoid compounds to cross biological membranes of the gastrointestinal tract and the blood–brain barrier by passive diffusion was investigated by the PAMPA model [23].

In the PAMPA-BBB experiments, only giffonin X (**3**) was detected in the acceptor phase. It also presented a calculated log*P_e_* value greater than −6.0 (−5.92 ± 0.04),which indicates that **3** is capable of crossing the lipid membrane of the blood–brain barrier (Table 6) [24]. However, compound **3** was considered unstable (*t*_1/2_ = 81.6 h) in the pH 7.4 medium of the PAMPA-BBB model and its decomposition product could not be detected in the acceptor phase.

In the PAMPA-GI model, compound **4** with one of the lowest *c*log*P* values (0.94 ± 0.46) among the studied diarylheptanoids was not detected in the acceptor phase, suggesting that it is unable to cross the lipid membrane of the gastrointestinal tract. Compounds **1**–**3** were detected in the acceptor phase in the PAMPA-GI model; however, none of the diarylheptanoids possessed log*P_e_* values greater than the critical −5.0 (Table 6), predicting that neither the compounds are able to pass through the membrane of the gastrointestinal tract [24]. 

Regarding the polarity of these constituents, none of the compounds have *c*log *P* values higher than 2.5. Compounds **2** and **3** have higher *c*log *P* values than 1.0, while *c*log *P* values of **1** and **4** are lower than 1.0. Compounds **1** and **2** are constitutional isomers; nevertheless, their *c*log *p* values are different (*c*log *P* 0.93 ± 0.46 and 1.92 ± 0.67, respectively). These data suggest poor membrane permeability of the major diarylheptanoid components of the *C. betulus* bark.

A further aspect to consider when assessing the PAMPA results is the decomposition of the constituents in aqueous media at the investigated pH values. Significant changes in compound concentrations occurring in a physiologically relevant time frame might be observed for **3** at pH 7.4 and **4** at pH 1.2. In these cases, the decrease in concentration in the donor and acceptor phases caused by decomposition of the analytes of interest might modify the PAMPA results.

The in vitro neuroprotective effect of cyclic diarylheptanoids in mouse hippocampal HT22 cells [25] and N2a cells [26] was established. However, based on our results suggesting poor penetration capability, their in vivo efficacy is ambiguous.

### 2.5. Evaluation of the Cytostatic Activity

The in vitro antiproliferative activities of the isolated *Carpinus* diarylheptanoids were studied by the Alamar Blue assay in HT-29 (colorectal carcinoma), HepG2 (hepatocellular carcinoma), HL-60 (acute promyelocytic leukaemia), U87 (glioblastoma) and A2058 (melanoma, derived from metastatic site: lymph node) human cancer cell lines for the first time (Supplementary Table S1). We confirmed the concentration-dependent antiproliferative activity of carpinontriol A (**1**) against A2058 human metastatic melanoma cells (IC_50_ = 14.9 ± 2.3 μM). It was comparable to that of the United States Food and Drug Administration (FDA)-approved etoposide (IC_50_ = 8.9 ± 0.2 μM). The cytostatic activity of **1** in A2058 cells was moderate when compared to the antitumor drug daunomycin (IC_50_ = 0.16 ± 2.3 μM). However, it should be noted that in contrast to daunomycin, compound **1** showed a highly selective antiproliferative activity.

No significant in vitro activity was observed for the other constituents at a concentration range of 0.16–100 μM. Our results are the following previous studies, since IC_50_ values exceeding 100 μM were observed for carpinontriol B (**2**) in A549 human lung adenocarcinoma and HeLa human cervical adenocarcinoma cells [17,27]. Similarly, carpinontriol B was not cytotoxic up to 1000 μM in A375 and SK-Mel-28 human melanoma cell lines [28].

## 3. Materials and Methods

### 3.1. Solvents and Chemicals

Chloroform, ethyl acetate and methanol of reagent grade as well as HPLC-grade methanol and acetonitrile were purchased from Molar Chemicals Kft. (Halásztelek, Hungary). Dimethyl sulfoxide (DMSO), *n*-dodecane, sodium chloride (NaCl), hydrochloric acid (HCl), disodium hydrogen phosphate heptahydrate (Na_2_HPO_4_∙7H_2_O) and sodium dihydrogen phosphate monohydrate (NaH_2_PO_4_∙H_2_O) were obtained from Reanal-Ker (Budapest, Hungary), while phosphatidylcholine, cholesterol and the porcine polar brain lipid extract were purchased from Merck (Darmstadt, Germany). Acetic acid 100% for HPLC LiChropur™, pyruvate and PBS tablet (Phosphate Buffered Saline, pH 7.4) were acquired from Sigma-Aldrich (Steinheim, Germany). Roswell Park Memorial Institute 1640 medium (RPMI-1640) and Dulbecco’s Modified Eagle’s Medium (DMEM) were supplied by Lonza (Basel, Switzerland). Fetal bovine serum (FBS) was purchased from Biosera (Nuaille, France). Non-essential amino acids, penicillin/streptomycin (10,000 units penicillin and 10 mg streptomycin/mL) and trypsin were obtained from Gibco (Thermo Fisher Scientific, Waltham, MA, USA). High-purity water was gained by a Millipore Direct Q5 Water Purification System (Billerica, MA, USA).

### 3.2. Plant Material and Sample Preparation

Bark samples of *C. betulus* were collected in Hungary, in the Visegrád Hills (Visegrádi-hegység, July 2018) to prepare the samples for the stability studies. Authenticated samples and herbarium specimens are deposited at the Herbarium of the Department of Pharmacognosy, Semmelweis University, Budapest, Hungary. Dried and milled samples (12 g) were extracted in an ultrasonic bath (Bandelin Sonorex Digitec DT 1028, Berlin, Germany) with chloroform, ethyl acetate and methanol consecutively (3 × 120 mL for all solvents, 2 h each) at room temperature. The extracts were distilled to dryness with a rotary evaporator (Büchi Rotavapor R-200, Flawil, Switzerland) at 45 °C. The samples were suspended in 70% methanol of HPLC-gradient grade and filtered through Minisart RC 15 0.2 µm syringe filters (Sartorius AG, Goettingen, Germany).

### 3.3. Isolation of Diarylheptanoids

For the isolation of the most dominant diarylheptanoids, *C. betulus* bark was collected in Mátraháza (Hungary; May 2016). Similarly to the analytical samples (Section 2.2), dried and milled bark (500 g) was extracted in an ultrasonic bath successively with chloroform, ethyl-acetate and methanol (3 × 2 L for all solvents, 2 h each). The methanol extract was evaporated to dryness under reduced pressure at 50 °C and suspended in 70% methanol (final concentration: 0.5 mg/mL). The extract was then fractionated by flash chromatography (CombiFlash NextGen 300+, Teledyne Isco, Lincoln, NE, USA), using a RediSep Rf Gold C18 column (100 g, Teledyne Isco) as stationary phase. Eluent A was 0.3% acetic acid in water; eluent B was methanol (gradient elution: 0 min 30% B, 4 min 62.5% B, 19 min 100% B, 29 min 100% B; flow rate: 60 mL/min). Fractions of 16 mL each were collected and further fractionated by (semi)preparative HPLC.

The combined fractions 31–34 were separated by semipreparative HPLC (Waters 2690 HPLC system equipped with Waters 996 diode array detector) (Waters Corporation, Milford, MA, USA). The Luna C18 100 A (150 × 10 mm i.d., 5 μm; Phenomenex Inc., Torrance, CA, USA) column as stationary phase, and 0.3% acetic acid in water (as eluent A) and methanol (as eluent B) were used. The following gradient elution was applied to obtain **1** (t_R_ = 22.3 min) and **4** (t_R_ = 30.0 min): 0 min 33% B, 20 min 33% B, 25 min 100% B, 33 min 100% B (flow rate: 1 mL/min).

Fractions 42–52 were combined and further chromatographed by preparative HPLC (Hanbon Sci.&Tech. Newstyle, Huaian, China) using a Gemini NX-C18 (150 × 21.2 mm, 5 μm; Phenomenex Inc.) column as stationary phase to collect 7 subfractions. The following gradient elution (flow rate: 5 mL/min) was used (eluent A: 0.3% acetic acid in water; eluent B: methanol): 0 min 40% B, 25 min 60% B, 26 min 100% B, 37 min 100% B. Subfraction 4 (t_R_ = 19 min) was separated using the same Waters 2690 HPLC instrument and Luna C18 100 A (150 × 10 mm i.d., 5 μm; Phenomenex Inc.) column as stationary phase. Eluent A was 0.3% acetic acid in water, eluent B was acetonitrile, flow rate of the mobile phase was 1 mL/min. The utilized gradient elution (0 min 35% B, 16 min 35% B, 17 min 100% B) yielded **2** (t_R_ = 14.0 min) and **3** (t_R_ = 14.8 min).

The quantity and purity of the isolated substances was as follows: carpinontriol A (**1**) (1.3 mg, >97%), carpinontriol B (**2**) (1.2 mg, >99%), giffonin X (**3**) (2.0 mg, >99%), 3,12,17-trihydroxytricyclo [12.3.1.1^2,6^]nonadeca-1(18),2(19),3,5,14,16-hexaene-8,11-dione (**4**) (1.0 mg, >98%). The purity of subfractions containing the isolated compounds collected during the final isolation step was evaluated by UHPLC-DAD (Supplementary Figures S14–S17).

### 3.4. UHPLC-DAD and UHPLC-DAD-HR-MS/MS Analyses

For the analysis of the samples from the chemical stability as well as the PAMPA studies, sample concentrations were determined using our previously developed ultrahigh-performance liquid chromatography–diode array detection (UHPLC-DAD) method validated for linearity, precision and accuracy [16]. Briefly, an ACQUITY UPLC H-Class PLUS System hyphenated with a quaternary solvent delivery pump (QSM), an auto-sampler manager (FTN), a column compartment (CM) and a photodiode array (PDA) detector (Waters Corporation) was employed. Stationary phase: Acquity BEH C18 column (100 × 2.1 mm i.d., 1.7 µm; Waters Corporation), column temperature: 30 °C. The mobile phase consisted of 0.3% acetic acid in water (eluent A) and acetonitrile (eluent B). The following gradient elution was applied at a flow rate of 0.3 mL/min: 0 min 12.0% B, 19.0 min 13.5% B, 25.5 min 75.0% B, 26.0 min 100.0% B, 28.0 min 100.0% B, 28.5 min 12.0% B. 

High-resolution mass spectra of the degradation products formed during the stability studies were obtained using a Dionex Ultimate 3000 UHPLC system (3000RS diode array detector, TCC-3000RS column thermostat, HPG-3400RS pump, SRD-3400 solvent rack degasser, WPS-3000TRS autosampler), hyphenated with an Orbitrap^®^ Q Exactive Focus Mass Spectrometer equipped with an electrospray ionization source (Thermo Fischer Scientific, Waltha, MA, USA). For the chromatographic separation of the constituents, the same Acquity UPLC BEH C18 (30 × 2.1 mm i.d., 1.7 μm; Waters Corporation) column as stationary phase (maintained at 25 °C) was used. Mobile phase: 0.1% formic acid in water (eluent A) and a mixture of 0.1% formic acid in water and acetonitrile (20:80, *v*/*v*) (eluent B). Gradient elution was as follows: 10–60% B (0.0–3.5 min), 60–100% B (3.5–4.0 min), 100% B (4.0–4.5 min), 100–10% B (4.5–7.0 min), flow rate: 0.3 mL/min. The ESI source was operated in negative ionization mode and operation parameters were optimized automatically using the built-in software. The working parameters were as follows: spray voltage 2500 V; capillary temperature 320 °C; sheath gas (N_2_), 47.5 °C; auxiliary gas (N_2_) 11.25 arbitrary units, spare gas (N_2_) 2.25 arbitrary units. The resolution of the full scan was of 70,000, the scanning range was between *m*/*z* 100–500 units. The most intense ions detected in full scan spectrum were selected for data-dependent MS/MS scan at a resolving power of 35,000, in the range of *m*/*z* 50–500. Parent ions were fragmented with normalized collision energy of 10%, 30% and 45%. 

### 3.5. Stability Studies

In the present work, we studied the effects of different conditions, including storage time, storage temperature and solvent, on the stability of the cyclic diarylheptanoids **1**–**4**. Their chemical stability at different pH values was also investigated. Additionally, degradation kinetics of the compounds were examined, while degradation pathways and mechanisms were also explored.

#### 3.5.1. Evaluation of Aqueous Stability at Different pH Values

The buffers modelling the gastric fluid (pH 1.2), the intestinal fluid (pH 6.8) and the blood and the tissues (pH 7.4) were prepared as follows. Buffer pH = 1.2: 1.0 g NaCl and 3.5 mL HCl dissolved in distilled water, final volume: 500.0 mL. Buffer pH = 6.8: 20.2 g Na_2_HPO_4_∙7H_2_O and 3.4 g NaH_2_PO_4_∙H_2_O dissolved in distilled water, final volume: 1000.0 mL, pH adjustment with 0.5 M NaOH or 0.5 M HCl. Buffer pH = 7.4: one PBS tablet dissolved in 200.0 mL distilled water. The stock solutions of compounds **1**–**4** were prepared with dimethyl sulfoxide (DMSO) at a concentration of 10.0 mM. The stock solutions were diluted 100-fold with each buffer separately to obtain the working solutions (297.0 μL buffer + 3.0 μL stock solution). All working solutions were filtered through Phenex-RC 15 mm, 0.2 μm syringe filters (Gen-Lab Ltd., Budapest, Hungary). The samples were incubated for 81 h at 37 °C; aliquots were taken for analysis every 9 h in accordance with the time required to quantify the analytes of interest in one set of samples. The total incubation time of 81 h was applied to obtain data for ten measurement points. The previously described UHPLC-DAD method was used to examine the changes in compound concentrations (see Section 3.4).

For the determination of pH stability, the initial AUC values were compared with the data after 9 and 81 h using paired-sample *t* test; significant difference was reported at *p* < 0.05. The effects of the pH were analysed through one-way analysis of variance (ANOVA) followed by Tukey’s post hoc HSD test (*p* < 0.05). All experiments were performed in triplicates (*n* = 3).

We used the following equations to calculate the first-order reaction rate constant (*k*) and the half-life (*t*_1/2_) indicating the time required to reduce the concentration of diarylheptanoids by 50% [29]:

ln (*c_t_*/*c*_0_) = −*k* × *t* (1)

*t*_1/2_ = −ln 0.5 × *k*^−1^ (2)

where *c_t_* is the concentration of the diarylheptanoids at time *t*, *c*_0_ is the initial concentration, *k* is the reaction rate constant, *t* is the treatment time. 

#### 3.5.2. Evaluation of Storage Stability

The chemical stability of the isolated compounds in solutions was examined at a concentration of 50 µg/mL in methanol and water (in the latter case using methanol as co-solvent, final composition: water-methanol 90:10, *v*/*v*). Furthermore, the methanol and ethyl acetate extracts of *C. betulus* bark (concentration 4 mg/mL) were also studied in order to assess the effects of the accompanying substances. The storage stability studies were performed at a neutral pH value. All solutions were filtered through Phenex-RC 15 mm, 0.2 μm syringe filters (Gen-Lab Ltd., Budapest, Hungary). The samples were prepared in triplicate and stored protected from light at 22 ± 2.0 °C, 5 ± 1.5 °C and −15 ± 2.0 °C for 23 weeks. Quantities of the analytes of interest were quantified at weeks 12 and 23 using the abovementioned UHPLC-DAD method (see Section 3.4).

For the determination of the stability, the initial AUC values were compared with the data of weeks 12 and 23 using paired-sample T test; significance was reported at *p* < 0.05. The effects of the temperature and the medium (i.e., solvent and accompanying substances) were analysed through one-way analysis of variance (ANOVA) followed by Tukey’s post hoc HSD test (*p* < 0.05). To establish the kinetic parameters *t*_1/2_ and *k*, Equations (1) and (2) were applied, respectively.

### 3.6. Parallel Artificial Membrane Permeability Assay (PAMPA) Studies

A parallel artificial membrane permeability assay (PAMPA) was used to determine the effective permeability (*Pe*) for the *Carpinus* diarylheptanoids. Stock solutions of the isolated compounds (10 mM in DMSO) were diluted with the defined buffer (pH 7.4 for the PAMPA-BBB and pH 6.8 for the PAMPA-GI assays) to obtain the donor solutions (composition: 297.0 μL buffer + 3.0 μL stock solution). Donor solutions were filtered through Phenex-RC 15 mm, 0.2 μm syringe filters (Gen-Lab Ltd., Budapest, Hungary).

For the PAMPA-BBB test, 5 μL of porcine polar brain lipid extract (PBLE) solution (16.0 mg PBLE + 8.0 mg cholesterol dissolved in 600.0 μL *n*-dodecane) was applied for each well of the 96-well polycarbonate-based filter donor plates (top plate) (Multiscreen™-IP, MAIPN4510, pore size 0.45 μm; Merck). For the PAMPA-GI assay, the wells of the top plate were coated with 5 μL of the mixture of 8.0 mg phosphatidylcholine + 4.0 mg cholesterol dissolved in 300.0 μL *n*-dodecane. The 150.0 μL aliquots of the filtrated donor solutions were placed on the membrane. The 96-well PTFE acceptor plates (bottom plates) (Multiscreen Acceptor Plate, MSSACCEPTOR; Merck), were filled with 300.0 μL buffer solution (0.01 M PBS buffer, pH 7.4). The donor plate was placed upon the acceptor plate, and both plates were incubated together at 37 °C for 4 h in a Heidolph Titramax 1000 Vibrating platform shaker (Heidolph, Schwabach, Germany).

After the incubation, the plates were separated and the compound concentrations in the donor (*C_D_*(*t*)) and acceptor (*C_A_*(*t*)) solutions were determined using the aforementioned UHPLC-DAD method (see Section 3.4). In advance, concentrations of the analytes of interest in the donor solutions at zero time point (*C_D_*(0)) were also established by UHPLC-DAD. The effective permeability and the membrane retention in the PAMPA-BBB and the PAMPA GI experiments were calculated by Equations (3) and (4), respectively [30]:

(3) $$P_{e}=\frac{-2.303}{A(t-\tau_{SS})}\cdot \left(\frac{V_{A}\cdot V_{D}}{V_{A}+V_{D}}\right)\cdot lg\left[1-\left(\frac{V_{A}+V_{D}}{\left(1-MR\right)\cdot V_{D}}\right)\times \left(\frac{C_{A}\left(t\right)}{C_{D}\left(0\right)}\right)\right]$$

(4) $$P_{e}=\frac{-2.303}{A(t-\tau_{SS)})}\cdot \left(\frac{1}{1+r_{a}}\right)\cdot lg\left[-r_{a}+\left(\frac{1+r_{a}}{1-MR}\right)\times \left(\frac{C_{D}\left(t\right)}{C_{D}\left(0\right)}\right)\right]$$

where *P_e_* is the effective permeability coefficient (cm/s), *A* is the filter area (0.24 cm^2^), *V_D_* and *V_A_* are the volumes in the donor (0.15 cm^3^) and acceptor phases (0.30 cm^3^), *t* is the incubation time (s), τ_SS_ is the time (s) to reach steady state (240 s), *C_D_*(*t*) is the concentration (mol/cm^3^) of the compound in the donor phase at time *t*, *C_D_*(0) is the concentration (mol/cm^3^) of the compound in the donor phase at time 0, MR is the estimated membrane retention factor (the estimated mole fraction of solute lost to the membrane) and *r_a_* is the sink asymmetry ratio (gradient-pH-induced), defined as:

(5) $$r_{a}=\frac{V_{D}}{V_{A}}\times \frac{P_{e}^{\left(A\to D\right)}}{P_{e}^{\left(D\to A\right)}}$$

(6) $$MR=1-\frac{C_{D}\left(t\right)}{C_{D}\left(0\right)}-\frac{V_{A}}{V_{D}}\frac{C_{A}\left(t\right)}{C_{D}\left(0\right)}$$

All experiments were performed in three triplicates on three consecutive days (*n* = 9); caffeine standard was used as positive, while rutin was used as negative control. *C*log *P* values were calculated using ACD/ChemSketch (Freeware) 2 January 2020 (Advanced Chemistry Development, Inc., Toronto, ON, Canada).

### 3.7. Evaluation of the In Vitro Activity of the Isolated Diarylheptanoids

#### 3.7.1. Cell Culturing and Media

For the experiments, the following human cell lines were used: A2058 (melanoma, derived from metastatic site: lymph node), HepG2 (hepatocellular carcinoma), U87 (glioblastoma), HT-29 (colorectal carcinoma) and HL-60 (acute promyelocytic leukaemia). Cell lines were generous gifts from Dr. József Tóvári (Department of Experimental Pharmacology, National Institute of Oncology, Budapest, Hungary).

For maintaining the U87 cell culture, DMEM supplemented with 10% FBS, 2 mM L-glutamine, 100 µg/mL penicillin/streptomycin, 1 mM pyruvate and 1% non-essential amino acids (CM DMEM) were used. A2058, HT-29, HepG2 and HL-60 cells were cultured in RPMI-1640 medium supplemented with 10% FBS, 2 mM L-glutamine and a penicillin-streptomycin antibiotics mixture (50 IU/mL and 50 μg/mL, respectively). The cultures were maintained at 37 °C in a humidified atmosphere with 5% CO_2_.

#### 3.7.2. Determination of the In Vitro Antiproliferative Activity

The cells were grown to confluency and then divided into 96-well tissue culture plates (Sarstedt, Nümbrecht, Germany) with an initial cell number of 5000 cells/well. Cells were incubated at 37 °C in a 5% CO_2_ humidified atmosphere overnight. Before the assay, 50 µL of the supernatant was removed and replaced with a 50 µL serum-free medium (SFM). The stock solutions of the compounds (c = 20 mM) were serially diluted with SFM and added to the cells in 100 μL volume. The final concentration of each compound in the cells was 0.16 µM, 0.8 µM, 4 µM, 20 µM and 100 µM (each concentration has four parallels). The cells were treated for 24 h with the compounds and negative control cells (no compound control) were treated with SFM only (incubated at 37 °C). As a positive control, we employed daunomycin (DAU) [31,32] and etoposide [33] as FDA-approved clinically used drugs as well as compound Sal (5-chloro-2-hydroxy-*N*-[4-(trifluoromethyl)phenyl]benzamide) as a cytostatic drug candidate [34]. After 24 h of incubation, cells were washed 3 times with SFM, and then the cells were further cultured in 10% FBS-containing complete medium (CM). After three days, a 22 µL Alamar Blue (resazurin sodium salt, Merck) solution (0.15 mg/mL in PBS) was added to each well, and after 4 h of incubation, the fluorescence was measured at λ_Ex_ = 530/30 and λ_Em_ = 610/10 nm using a Synergy H4 multi-mode microplate reader (BioTek, Bad Friedrichshall, Germany). The percentage of cytostasis was calculated with the following equation:

Cytostatic effect (%) = [1 − (OD_treated_/OD_control_)] × 100 (7)

where the values OD_treated_ and OD_control_ correspond to the optical densities of the treated and the control wells, respectively.

Cytostasis (%) was plotted as a function of concentration, fitted to a dose–response curve and the 50% inhibitory concentration (IC_50_) value was determined from these curves. Data were evaluated with Excel (version: 365; Microsoft, Redmond, WA, USA) and the curves were defined using Microcal OriginPro (version: 2018; OriginLab, Northampton, MA, USA) software.

In each case, two independent experiments were carried out with four parallel measurements and the mean IC_50_ values together with ±SD were represented. The Excel (version: 365) (Microsoft, Redmond, WA, USA) and Microcal OriginPro (version: 2018) (OriginLab, Northampton, MA, USA) softwares were used for data evaluation.

## 4. Conclusions

In the present work, we isolated the most characteristic *meta*,*meta*-cyclophane-type diarylheptanoids from the bark of the European hornbeam: carpinontriols A (**1**) and B (**2**), giffonin X (**3**) and 3,12,17-trihydroxytricyclo [12.3.1.1^2,6^]nonadeca-1(18),2(19),3,5,14,16-hexaene-8,11-dione (**4**).

Stability testing is essential in the development of new pharmaceuticals. Therefore, we investigated the effects of ambient conditions, including storage time, temperature and medium (pH, solvent and accompanying constituents), on the degradation of *Carpinus* diarylheptanoids **1**–**4**. Degradation kinetics of the cyclic diarylheptanoid compounds were also examined. No significant decrease in the concentration was observed and no degradation products were detected for carpinontriol B (**2**); therefore, it was considered as stable under all investigated conditions. Compound **4** was susceptible of decomposing only at acidic pH values, while the storage time, the temperature and the medium did not affect its concentration. On the other hand, carpinontriol A (**1**) and giffonin X (**3**) showed significant decomposition, and degradation products were also detected in their UHPLC-HR-MS chromatograms. Degradation pathways of **1** and **3** were explored and degradation mechanisms involving the cleavage of a water molecule were proposed for them.

The membrane penetration ability of the isolated compounds was also studied by the PAMPA method. Compounds **1**–**3** were all detected in the acceptor phase in the PAMPA-GI model; however, their log*P_e_* values being lower than −5.0 pointed to a poor membrane permeability. On the other hand, only giffonin X (**3**) was detected in the acceptor phase in the PAMPA-BBB model, and its log*P_e_* value (−5.92 ± 0.04) also suggested that it is capable of crossing the lipid membrane. Nonetheless, calculated *c*log *P* values of all compounds were lower than 2.5, indicating that they are indeed not able to cross biological membranes by passive diffusion.

The antiproliferative activity of the compounds was evaluated by the Alamar Blue assay in human HT-29 colon cancer, HepG2 hepatocellular carcinoma, HL-60 leukaemia, U87 glioblastoma and A2058 metastatic melanoma cells to obtain a dose–response for the new compounds. The highly selective cytostatic activity of carpinontriol A (**1**) in human metastatic melanoma cells was reported for the first time (IC_50_ = 14.9 ± 2.3 µM). Furthermore, similar activity to the etoposide control (IC_50_ = 8.9 ± 0.2 µM) was obtained on the A2058 cell type for compound **1**. 

## Figures and Tables

**Table 1:** Aqueous stability of the isolated diarylheptanoid compounds ^a,b^.

Incubation Time	pH	Compound			
		1	2	3	4
9 h	pH 7.4	96.9 ± 1.2 ^a^	102.0 ± 0.9 ^a^	82.6 ± 7.7 ^#b^	102.0 ± 3.7 ^a^
	pH 6.8	97.4 ± 1.5 ^#a^	105.2 ± 3.9 ^a^	98.9 ± 0.8 ^a^	105.8 ± 2.6 ^a^
	pH 1.2	97.1 ± 8.9 ^a^	101.7 ± 4.2 ^a^	100.6 ± 6.9 ^a^	68.5 ± 4.5 ^#^
81 h	pH 7.4	71.5 ± 5.2 ^#b^	101.5 ± 1.1 ^a^	46.7 ± 4.7 ^#^	103.1 ± 2.2 ^a^
	pH 6.8	75.3 ± 3.0 ^#b^	101.9 ± 6.4 ^a^	93.2 ± 2.0 ^#ab^	88.9 ± 2.0 ^#^
	pH 1.2	70.5 ± 2.6 ^#b^	100.7 ± 5.1 ^a^	83.4 ± 5.3 ^#b^	31.0 ± 7.0 ^#^

^a^ Data are expressed as relative concentrations (%) after 9 and 81 h compared to the initial value. ^b^ Data are expressed as mean values ± SD (*n* = 3). Values with identical lower-case letters (a–b) in the same column are not significantly different (Tukey test, *p* < 0.05); ^#^ *p* < 0.05 compared with the initial concentration.

**Table 2:** Kinetic parameters of the investigated *Carpinus* diarylheptanoids following storage at 37 °C at different pH values.

Compound	pH 7.4		pH 6.8		pH 1.2	
	*k* (h^−1^)	*t*_1/2_ (h)	*k* (h^−1^)	*t*_1/2_ (h)	*k* (h^−1^)	*t*_1/2_ (h)
**1**	4.19 × 10^−3^	165.6	3.65 × 10^−3^	189.6	4.15 × 10^−3^	167.1
**2**	-	-	-	-	-	-
**3**	8.49 × 10^−3^	81.6	8.38 × 10^−4^	826.8	2.32 × 10^−3^	298.4
**4**	-	-	1.42 × 10^−3^	487.7	1.27 × 10^−2^	54.4

**Table 3:** Chemical stability of *Carpinus* diarylheptanoids: Effects of storage time, temperature and medium on the concentrations of compounds **1**–**4** as compared to the initial value (%) ^a^.

Storage Time (Week)	Storage Temperature (°C)	Medium	Compound			
			1	2	3	4
12	22	SM	63.5 ± 8.7 ^#a^	99.7 ± 0.4 ^a^	84.5 ± 7.4 ^#a^	100.1 ± 0.5 ^ab^
		SA	61.7 ± 5.7 ^#a^	100.4 ± 1.1 ^a^	96.2 ± 0.1 ^#bcd^	100.8 ± 1.1 ^a^
		EE	110.9 ± 3.6 ^#bc^	100.2 ± 0.7 ^a^	101.6 ± 2.2 ^#bc^	106.3 ± 2.6 ^#^
		EM	114.0 ± 2.2 ^#b^	101.1 ± 1.1 ^a^	102.7 ± 0.9 ^#bc^	120.9 ± 2.6 ^#^
	5	SM	90.8 ± 2.3 ^#def^	99.4 ± 0.6 ^a^	99.0 ± 0.1 ^#bcd^	100.1 ± 0.6 ^ab^
		SA	91.4 ± 0.2 ^#def^	99.9 ± 0.4 ^a^	99.3 ± 0.1 ^#bcd^	100.5 ± 0.7 ^ab^
		EE	104.6 ± 1.0 ^#bcg^	99.5 ± 0.4 ^#a^	100.8 ± 0.5 ^#bcd^	102.0 ± 1.7
		EM	110.1 ± 6.2 ^bc^	99.9 ± 0.8 ^a^	100.2 ± 1.1 ^bcd^	105.5 ± 2.5
	−15	SM	94.0 ± 0.8 ^#efg^	99.1 ± 1.3 ^a^	100.4 ± 1.2 ^bcd^	99.1 ± 0.7 ^ab^
		SA	93.5 ± 0.4 ^#ef^	99.1 ± 1.3 ^a^	100.1 ± 0.6 ^bcd^	98.6 ± 1.3 ^b^
		EE	100.5 ± 0.5 ^ceg^	100.1 ± 0.7 ^a^	103.5 ± 0.2 ^b^	103.5 ± 0.8
		EM	108.7 ± 1.5 ^#bc^	99.8 ± 0.7 ^a^	101.6 ± 3.1 ^#bc^	102.8 ± 3.3 ^#^
23	22	SM	32.3 ± 6.3 ^#h^	99.9 ± 0.6 ^a^	67.1 ± 7.5 ^#^	100.2 ± 0.5 ^ab^
		SA	23.0 ± 0.5 ^#h^	99.8 ± 0.9 ^a^	87.6 ± 0.7 ^#ae^	100.3 ± 0.6 ^ab^
		EE	81.5 ± 3.9 ^#di^	99.7 ± 0.5 ^a^	95.7 ± 0.5 ^#cd^	108.5 ± 2.7 ^#^
		EM	77.7 ± 2.9 ^#ij^	99.7 ± 0.3 ^#a^	95.6 ± 2.4 ^#cd^	135.7 ± 9.7 ^#^
	5	SM	67.1 ± 7.5 ^#aj^	99.2 ± 0.5 ^a^	81.9 ± 1.0 ^#a^	100.3 ± 0.5 ^ab^
		SA	87.2 ± 0.4 ^#dfi^	99.9 ± 0.5 ^a^	99.1 ± 0.1 ^#bcd^	100.0 ± 0.5 ^ab^
		EE	92.1 ± 1.3 ^#def^	100.1 ± 0.4 ^a^	100.9 ± 1.3 ^bcd^	107.8 ± 2.6 ^#^
		EM	84.1 ± 1.1 ^#dfi^	99.2 ± 1.2 ^a^	93.8 ± 1.3 ^#de^	107.0 ± 4.1
	−15	SM	88.9 ± 3.1 ^#dfi^	99.1 ± 1.2 ^a^	95.8 ± 0.1 ^#cd^	99.8 ± 1.2 ^ab^
		SA	93.7 ± 0.3 ^#efg^	99.1 ± 1.2 ^a^	97.9 ± 2.9 ^bcd^	99.9 ± 1.2 ^ab^
		EE	91.3 ± 4.2 ^#def^	99.8 ± 0.4 ^a^	101.1 ± 2.7 ^#bcd^	108.0 ± 2.2 ^#^
		EM	88.3 ± 3.4 ^#dfi^	99.6 ± 0.7 ^a^	97.6 ± 3.2 ^bcd^	103.6 ± 3.5

^a^ Results are expressed as mean values ± SD (*n* = 3). Values with identical lower-case letters (a–j) in the same column are not significantly different (Tukey test, *p* < 0.05); ^#^ *p* < 0.05 compared with the initial samples. Abbreviations: SM: methanol solution; SA: aqueous solution; EE: ethyl acetate extract; EM: methanol extract.

**Table 4:** Kinetic parameters of carpinontriol A (**1**) and giffonin X (**3**) in aqueous and methanolic solutions following storage at 22 °C, 5 °C and −15 °C for 23 weeks.

Temperature (°C)	Medium	Carpinontriol A (1)		Giffonin X (3)	
		*k* (Week^−1^)	*t*_1/2_ (Week)	*k* (Week^−1^)	*t*_1/2_ (Week)
22	SA	5.97 × 10^−2^	11.61	5.90 × 10^−3^	117.48
	SM	4.53 × 10^−2^	15.30	1.85 × 10^−2^	37.47
5	SA	4.47 × 10^−3^	147.48	5.0 × 10^−4^	1386.29
	SM	1.40 × 10^−2^	50.23	7.10 × 10^−3^	97.63
−15	SA	1.70 × 10^−3^	407.73	-	-
	SM	3.60 × 10^−3^	192.54	1.60 × 10^−3^	433.22

Abbreviations: SA: aqueous solution; SM: methanol solution.

**Table 6:** Results of the PAMPA experiments: log*P_e_* values (*n* = 9) and the calculated *c*log *P* values (Chemsketch Freeware).

Compound	log*P_e_* PAMPA-BBB (*n* = 9)	log*P_e_* PAMPA-GI (*n* = 9)	*c*log *P*
**1**	n.d.	−6.25 ± 0.04	0.93 ± 0.46
**2**	n.d.	−5.46 ± 0.06	1.92 ± 0.67
**3**	−5.92 ± 0.04	−5.22 ± 0.07	1.77 ± 0.41
**4**	n.d.	n.d.	0.94 ± 0.46

Abbreviations: n.d.: not detected in the acceptor phase; PAMPA-GI: parallel artificial membrane permeability assay for the gastrointestinal tract; PAMPA-BBB: parallel artificial membrane permeability assay for the blood–brain barrier.

## Data Availability

Not applicable.

## Supplementary Materials

The following supporting information can be downloaded at: https://www.mdpi.com/article/10.3390/ijms241713489/s1.

## References

[1] Dai G., Tong Y., Chen X., Ren Z., Ying X., Yang F., Chai K. (2015). Myricanol Induces Apoptotic Cell Death and Anti-Tumor Activity in Non-Small Cell Lung Carcinoma in Vivo. Int. J. Mol. Sci..

[2] Tang G., Dong X., Huang X., Huang X.J., Liu H., Wang Y., Ye W.C., Shi L. (2015). A Natural Diarylheptanoid Promotes Neuronal Differentiation via Activating ERK and PI3K-Akt Dependent Pathways. Neurosci..

[3] Vanucci-Bacqué C., Bedos-Belval F. (2021). Anti-Inflammatory Activity of Naturally Occuring Diarylheptanoids – A Review. Bioorg. Med. Chem..

[4] Shen S., Liao Q., Feng Y., Liu J., Pan R., Lee S.M.-Y., Lin L. (2019). Myricanol Mitigates Lipid Accumulation in 3T3-L1 Adipocytes and High Fat Diet-Fed Zebrafish via Activating AMP-Activated Protein Kinase. Food Chem..

[5] Jahng Y., Park J.G. (2018). Recent Studies on Cyclic 1,7-Diarylheptanoids: Their Isolation, Structures, Biological Activities, and Chemical Synthesis. Molecules.

[6] Alberti Á., Riethmüller E., Béni S. (2018). Characterization of Diarylheptanoids: An Emerging Class of Bioactive Natural Products. J. Pharm. Biomed. Anal..

[7] Lv H., She G. (2012). Naturally Occurring Diarylheptanoids—A Supplementary Version. Rec. Nat. Prod..

[8] Doello K., Ortiz R., Alvarez P.J., Melguizo C., Cabeza L., Prados J. (2018). Latest in Vitro and in Vivo Assay, Clinical Trials and Patents in Cancer Treatment using Curcumin: A Literature Review. Nutr. Cancer.

[9] Nelson K.M., Dahlin J.L., Bisson J., Graham J., Pauli G.F., Walters M.A. (2017). The Essential Medicinal Chemistry of Curcumin. J. Med. Chem..

[10] Lee S.G., Shin D.J., Lee E.S., Goo Y.T., Kim C.H., Yoon H.Y., Lee M.W., Bang H., Seo S.J., Choi Y.W. (2018). Enhanced Chemical Stability of Hirsutenone Incorporated into a Nanostructured Lipid Carrier Formulation Containing Antioxidants. Bull. Korean Chem. Soc..

[11] Moon K.Y., Ahn B.K., Lee S.G., Lee S.H., Yeom D.W., Choi Y.W. (2011). Enhanced Aqueous Stability of Hirsutenone with Antioxidant. J. Pharm. Investig..

[12] Felegyi-Tóth C.A., Tóth Z., Garádi Z., Boldizsár I., Nedves A.N., Simon A., Felegyi K., Alberti Á., Riethmüller E. (2022). Membrane Permeability and Aqueous Stability Study of Linear and Cyclic Diarylheptanoids from *Corylus maxima*. Pharmaceutics.

[13] Lee J.S., Kim H.J., Park H., Lee Y.S. (2002). New Diarylheptanoids from the Stems of *Carpinus cordata*. J. Nat. Prod..

[14] Masullo M., Lauro G., Cerulli A., Bifulco G., Piacente S. (2022). *Corylus avellana*: A Source of Diarylheptanoids With α-Glucosidase Inhibitory Activity Evaluated by in vitro and in silico Studies. Front. Plant Sci..

[15] Masullo M., Lauro G., Cerulli A., Kontek B., Olas B., Bifulco G., Piacente S., Pizza C. (2021). Giffonins, Antioxidant Diarylheptanoids from *Corylus avellana*, and Their Ability to Prevent Oxidative Changes in Human Plasma Proteins. J. Nat. Prod..

[16] Felegyi-Tóth C.A., Garádi Z., Darcsi A., Csernák O., Boldizsár I., Béni S., Alberti Á. (2022). Isolation and Quantification of Diarylheptanoids from European Hornbeam (*Carpinus betulus* L.) and HPLC-ESI-MS/MS Characterization of Its Antioxidative Phenolics. J. Pharm. Biomed. Anal..

[17] Cerulli A., Lauro G., Masullo M., Cantone V., Olas B., Kontek B., Nazzaro F., Bifulco G., Piacente S. (2017). Cyclic Diarylheptanoids from *Corylus avellana* Green Leafy Covers: Determination of Their Absolute Configurations and Evaluation of Their Antioxidant and Antimicrobial Activities. J. Nat. Prod..

[18] Liu J.-X., Di D.-L., Wei X.-N., Han Y. (2008). Cytotoxic Diarylheptanoids from the Pericarps of Walnuts (*Juglans regia*). Planta Med..

[19] Dai G., Tong Y., Chen X., Ren Z., Yang F. (2015). In vitro Anticancer Activity of Myricanone in Human Lung Adenocarcinoma A549 Cells. Chemotherapy.

[20] Xue Y., Savchenko A.I., Agnew-Francis K.A., Miles J.A., Holt T., Lu H., Chow S., Forster P.I., Boyle G.M., Ross B.P. (2023). seco-Pregnane Glycosides from Australian Caustic Vine (*Cynanchum viminale* subsp. australe). J. Nat. Prod..

[21] Friedman M., Jürgens H.S. (2000). Effect of pH on the Stability of Plant Phenolic Compounds. J. Agric. Food Chem..

[22] Tabboon P., Tuntiyasawasdikul S., Sripanidkulchai B. (2019). Quality and Stability Assessment of Commercial Products Containing Phytoestrogen Diaryheptanoids from *Curcuma comosa*. Ind. Crops Prod..

[23] Ayanlowo A.G., Garádi Z., Boldizsár I., Darcsi A., Nedves A.N., Varjas B., Simon A., Alberti Á., Riethmüller E. (2020). UHPLC-DPPH Method Reveals Antioxidant Tyramine and Octopamine Derivatives in *Celtis occidentalis*. J. Pharm. Biomed. Anal..

[24] Könczöl Á., Müller J., Földes E., Béni Z., Végh K., Kéry Á., Balogh G.T. (2013). Applicability of a Blood–Brain Barrier Specific Artificial Membrane Permeability Assay at the Early Stage of Natural Product-Based CNS Drug Discovery. J. Nat. Prod..

[25] Kim N.T., Lee D.S., Chowdhury A., Lee H., Cha B.Y., Woo J.T., Woo E.R., Jang J.H. (2017). Acerogenin C from *Acer nikoense* exhibits a neuroprotective effect in mouse hippocampal HT22 cell lines through the upregulation of Nrf-2/HO-1 signaling pathways. Mol Med Rep.

[26] Chen P., Lin X., Yang C.-H., Tang X., Chang Y.-W., Zheng W., Luo L., Xu C., Chen Y.-H. (2017). Study on Chemical Profile and Neuroprotective Activity of *Myrica rubra* Leaf Extract. Molecules.

[27] Masullo M., Cerulli A., Mari A., de Souza Santos C.C., Pizza C., Piacente S. (2017). LC-MS Profiling Highlights Hazelnut (Nocciola di Giffoni PGI) Shells as a Byproduct Rich in Antioxidant Phenolics. Food Res. Int..

[28] Esposito T., Sansone F., Franceschelli S., Del Gaudio P., Picerno P., Aquino R.P., Mencherini T. (2017). Hazelnut (*Corylus avellana* L.) Shells Extract: Phenolic Composition, Antioxidant Effect and Cytotoxic Activity on Human Cancer Cell Lines. Int. J. Mol. Sci..

[29] Pacheco-Palencia L.A., Hawken P., Talcott S.T. (2007). Phytochemical, Antioxidant and Pigment Stability of Açai (*Euterpe oleracea* Mart.) as Affected by Clarification, Ascorbic Acid Fortification and Storage. Food Res. Int..

[30] Avdeef A. (2012). Permeability—PAMPA. Absorption and Drug Development.

[31] Gewirtz D. (1999). A Critical Evaluation of the Mechanisms of Action Proposed for the Antitumor Effects of the Anthracycline Antibiotics Adriamycin and Daunorubicin. Biochem. Pharmacol..

[32] Lajkó E., Spring S., Hegedüs R., Biri-Kovács B., Ingebrandt S., Mező G., Kőhidai L. (2018). Comparative Cell Biological Study of in vitro Antitumor and Antimetastatic Activity on Melanoma Cells of GnRH-III-containing Conjugates Modified with Short-chain Fatty Acids. Beilstein J. Org. Chem..

[33] Meresse P., Dechaux E., Monneret C., Bertounesque E. (2004). Etoposide: Discovery and Medicinal Chemistry. Curr. Med. Chem..

[34] Baranyai Z., Biri-Kovács B., Krátký M., Szeder B., Debreczeni M.L., Budai J., Kovács B., Horváth L., Pári E., Németh Z. (2021). Cellular Internalization and Inhibition Capacity of New Anti-Glioma Peptide Conjugates: Physicochemical Characterization and Evaluation on Various Monolayer- and 3D-Spheroid-Based in Vitro Platforms. J. Med. Chem..

