# Supplementary material for: Evaluation of the Chemical Stability, Membrane Permeability and Antiproliferative Activity of Cyclic Diarylheptanoids from European Hornbeam (Carpinus betulus L.)
Source: Int J Mol Sci. 2023 Aug 30;24(17):13489. doi: 10.3390/ijms241713489 (PMC10488193; doi:10.3390/ijms241713489)
Supplement: Supplementary file 1 [file ijms-24-13489-s001.zip › ijms-2556447-supplementary.pdf]

Supplementary Material

Evaluation of the Chemical Stability, Membrane Permeability and Antiproliferative Activity of Cyclic Diarylheptanoids from European Hornbeam (*Carpinus betulus* L.)

Csenge Anna Felegyi-Tóth [1], Tímea Heilmann [1], Eszter Buda [1], Bence Stipsicz [2,3], Alexandra Simon [1], Imre Boldizsár [1,4], Szilvia Bősze [3,5], Eszter Riethmüller [1] and Ágnes Alberti [1,*]

[1] Department of Pharmacognosy, Semmelweis University, Üllői út 26, 1085 Budapest, Hungary; felegyi_toth.csenge_anna@pharma.semmelweis-univ.hu (C.A.F.-T.); heilmann.timea@stud.semmelweis.hu (T.H.); buda.eszter@stud.semmelweis.hu (E.B.); simon.alexandra@pharma.semmelweis-univ.hu (A.S.); boldizsar.imre@semmelweis.hu (I.B.); riethmuller.eszter@pharma.semmelweis-univ.hu (E.R.)
[2] Institute of Biology, Doctoral School of Biology, Eötvös Loránd University, Pázmány Péter sétány 1/C, H-1117 Budapest, Hungary; stipsicz@student.elte.hu
[3] ELKH-ELTE Research Group of Peptide Chemistry, Eötvös Loránd Research Network, Eötvös Loránd University, Pázmány Péter sétány 1/A, H-1117 Budapest, Hungary; szilvia.bosze@ttk.elte.hu
[4] Department of Plant Anatomy, Institute of Biology, Eötvös Loránd University, Pázmány Péter sétány 1/C, 1117 Budapest, Hungary
[5] National Public Health Center, Albert Flórián út 2-6, 1097 Budapest, Hungary
* Correspondence: alberti.agnes@semmelweis.hu

Table of Contents

Table S1. Effect of *C. betulus* diarylheptanoids on human cancer cell lines

Figure S1. Chemical stability of carpinontriol A (**1**) and giffonin X (**3**) as a function of time, temperature and solvent

Figure S2. HR-ESI-MS spectrum of compound **1**

Figure S3. HR-MS/MS spectrum of compound **1**

Figure S4. HR-ESI-MS spectrum of compound **1a**

Figure S5. HR-MS/MS spectrum of compound **1a**

Figure S6. HR-ESI-MS spectrum of compound **1b**

Figure S7. HR-MS/MS spectrum of compound **1b**

Figure S8. HR-ESI-MS spectrum of compound **1c**

Figure S9. HR-MS/MS spectrum of compound **1c**

Figure S10. HR-ESI-MS spectrum of compound **3**

Figure S11. HR-MS/MS spectrum of compound **3**

Figure S12. HR-ESI-MS spectrum of compound **3a**

Figure S13. HR-MS/MS spectrum of compound **3a**

Figure S14. UHPLC-DAD chromatogram of a fraction containing **1**

Figure S15. UHPLC-DAD chromatogram of a fraction containing **2**

Figure S16. UHPLC-DAD chromatogram of a fraction containing **3**

Figure S17. UHPLC-DAD chromatogram of a fraction containing **4**

Table S1. Effect of *C. betulus* diarylheptanoids on human cancer cell lines[a]

**Compound**	**Cell Line[b]**				
	**HT-29 IC_{50} ± SD (μM)[c]**	**HepG2 IC_{50} ± SD (μM)**	**HL-60 IC_{50} ± SD (μM)**	**U87 IC_{50} ± SD (μM)**	**A2058 IC_{50} ± SD (μM)**
**1**	> 100	> 100	> 100	> 100	14.9 ± 2.3
**2**	> 100	> 100	> 100	> 100	> 100
**3**	> 100	> 100	> 100	> 100	> 100
**4**	> 100	> 100	> 100	> 100	> 100
**Reference compounds[d]**					
**Etoposide**	18.5 ± 1.7[29]	20.9 ± 1.2[29]	no data	27.0 ± 2.3[29]	8.9 ± 0.2[29]
**Dau**	0.2 ± 0.01[30]	1.2 ± 0.2[30]	0.02 ± 0.01[30]	0.4± 0.05[30]	0.16 ± 0.1[30]
**Sal**	no data	5.8± 0.7[30]	4.5 ± 0.7	0.8 ± 0.330	6.8 ± 1.2

[a] Data are expressed as means ± SD (n = 2). [b] HT-29 colorectal carcinoma cells, HepG2 hepatocellular carcinoma cells, HL-60 acute promyelocytic leukaemia cells, U87 glioblastoma cells, A2058 metastatic melanoma cells. [c] IC_{50}: required concentration of compounds to inhibit cell proliferation by 50% expressed as μM; [d] Experimental data in agreement with previous literature data. Abbreviations: Dau: Daunomycin; Sal: 5-chloro-2-hydroxy-*N*-[4-(trifluoromethyl)phenyl]benzamide, MW: 315.7 g/mol.

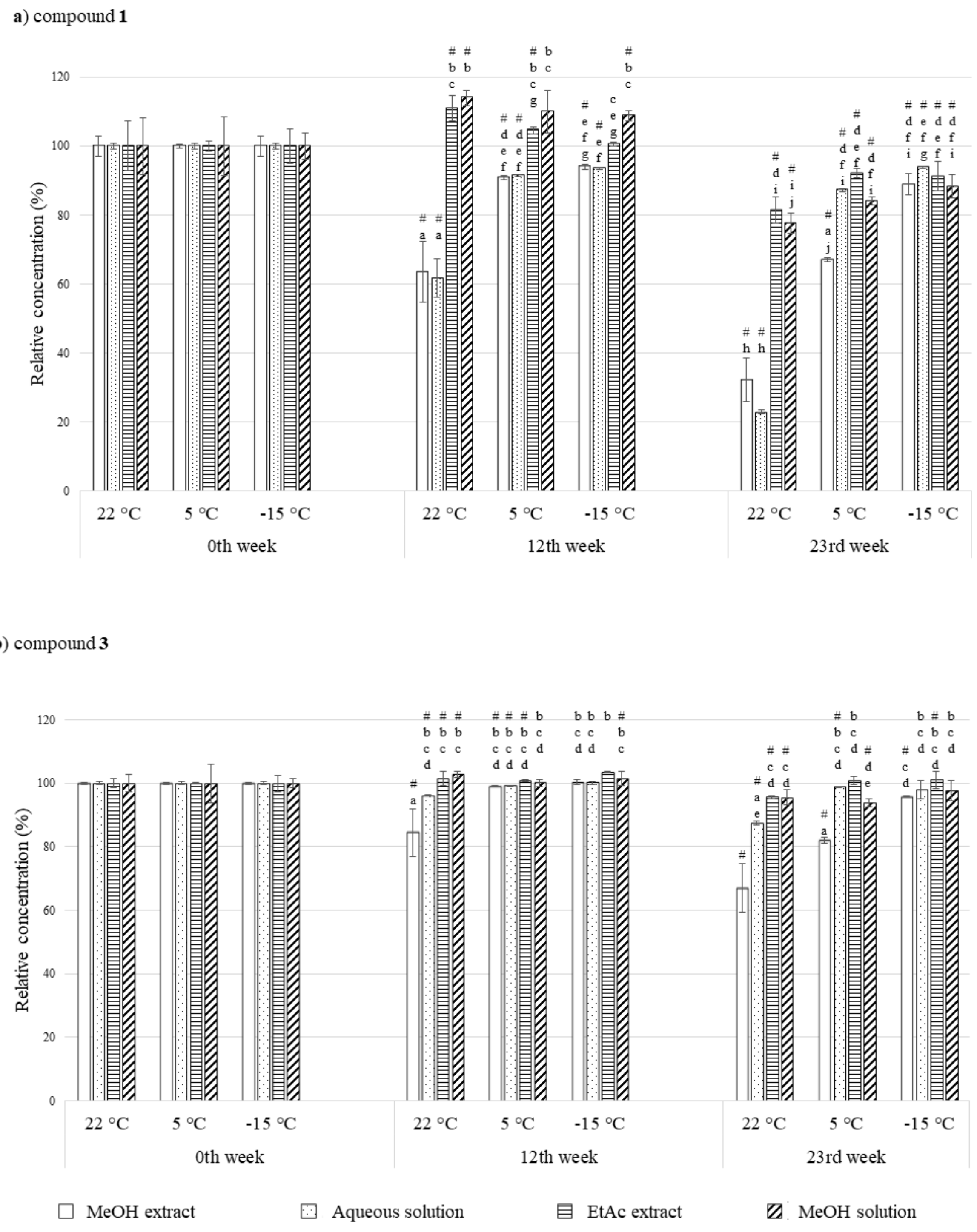

Figure S1. Chemical stability of carpinontriol A (**1**) and giffonin X (**3**) as a function of time, temperature and solvent. Concentrations of compounds are relative (%) values compared to the initial value. Results are expressed as mean values ± SD (n = 3). Values for individual compounds with identical lower case letters (a–j) are not significantly different (Tukey test, $p < 0.05$); $^{\#}$ $p < 0.05$ compared with the initial samples. Abbreviations: MeOH: methanol; EtAc: ethyl acetate.

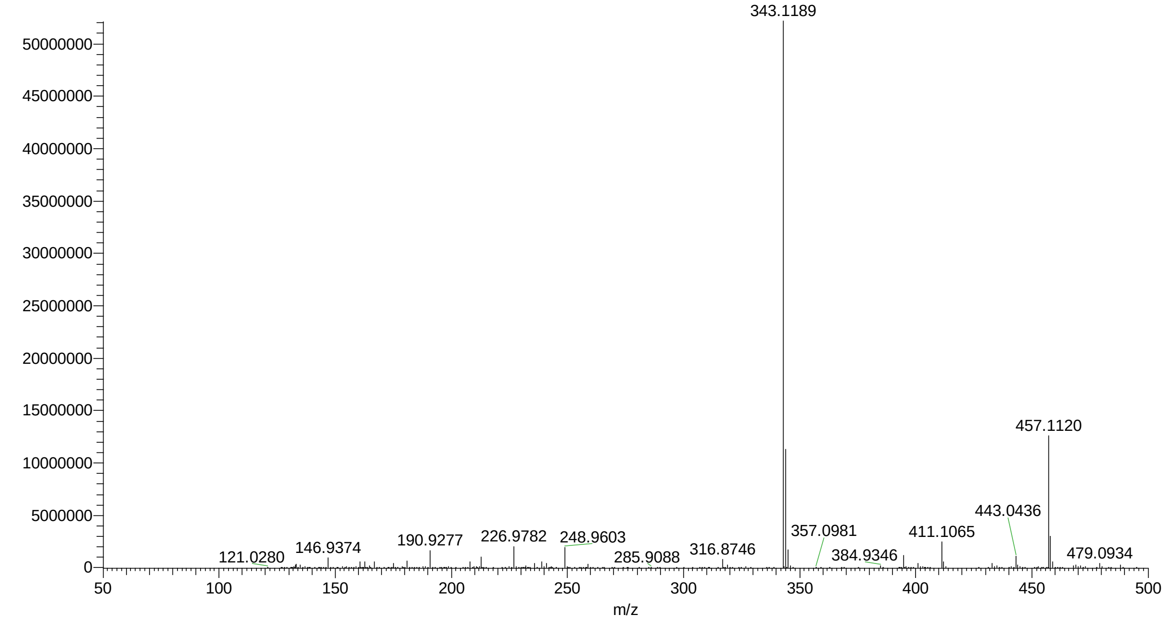

Figure S2. HR-ESI-MS spectrum of compound **1**

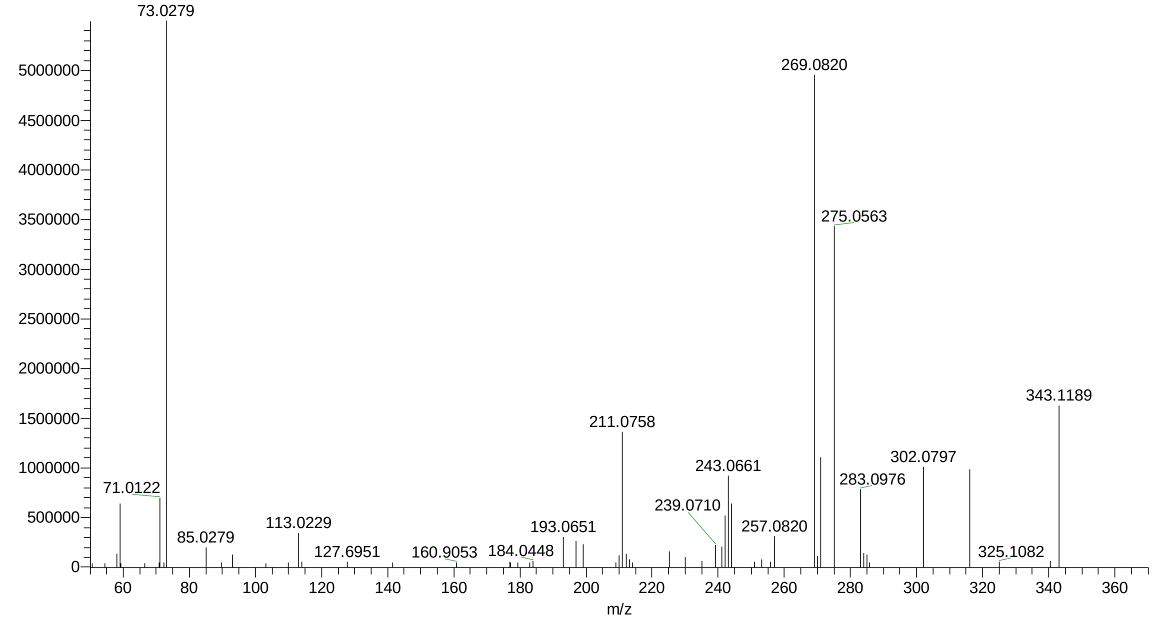

Figure S3. HR-MS/MS spectrum of compound **1**

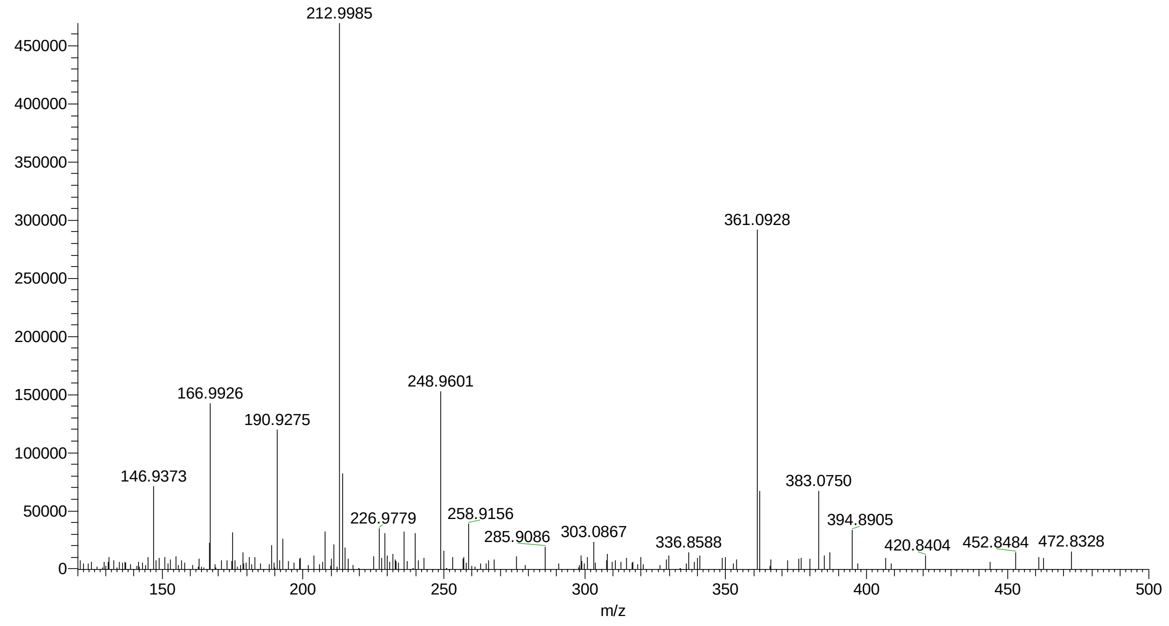

Figure S4. HR-ESI-MS spectrum of compound **1a**

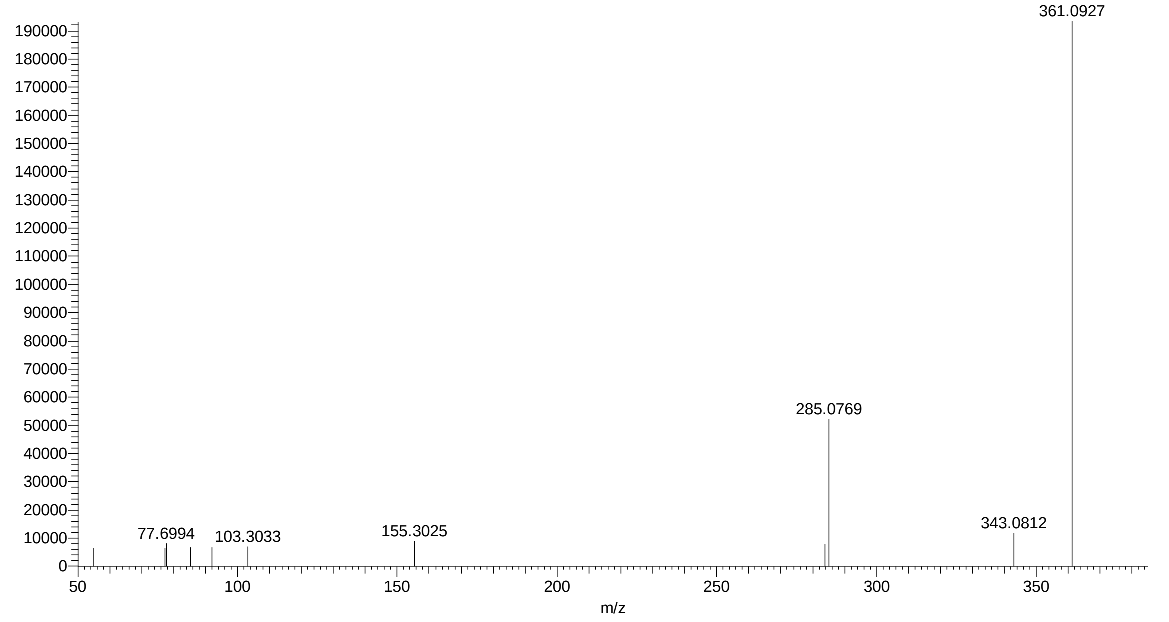

Figure S5. HR-MS/MS spectrum of compound **1a**

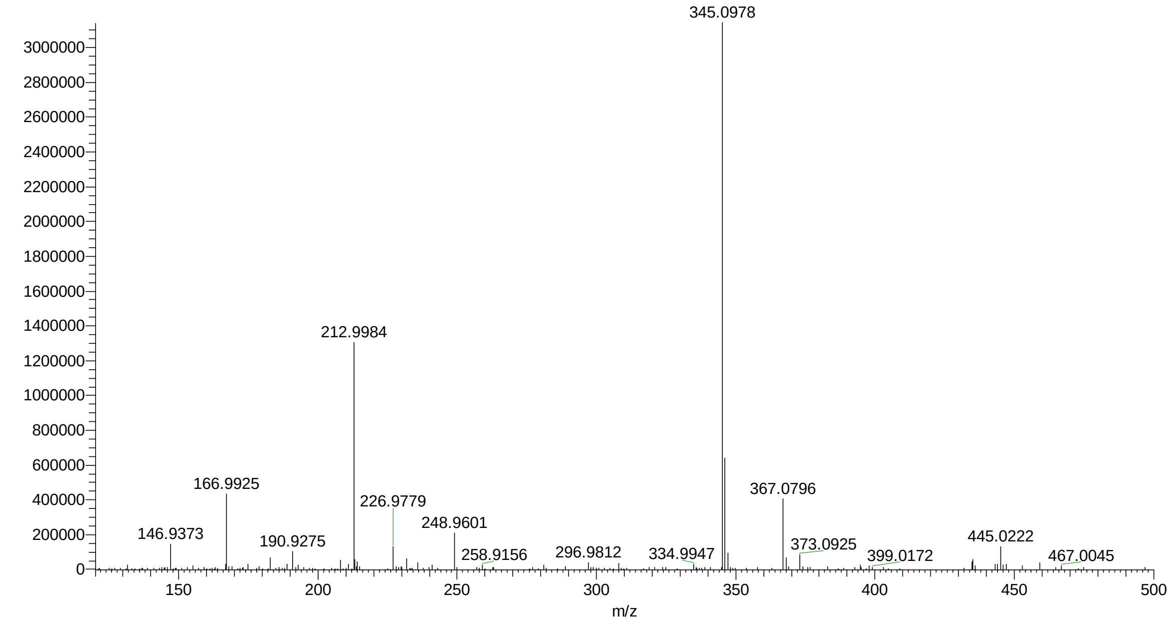

Figure S6. HR-ESI-MS spectrum of compound **1b**

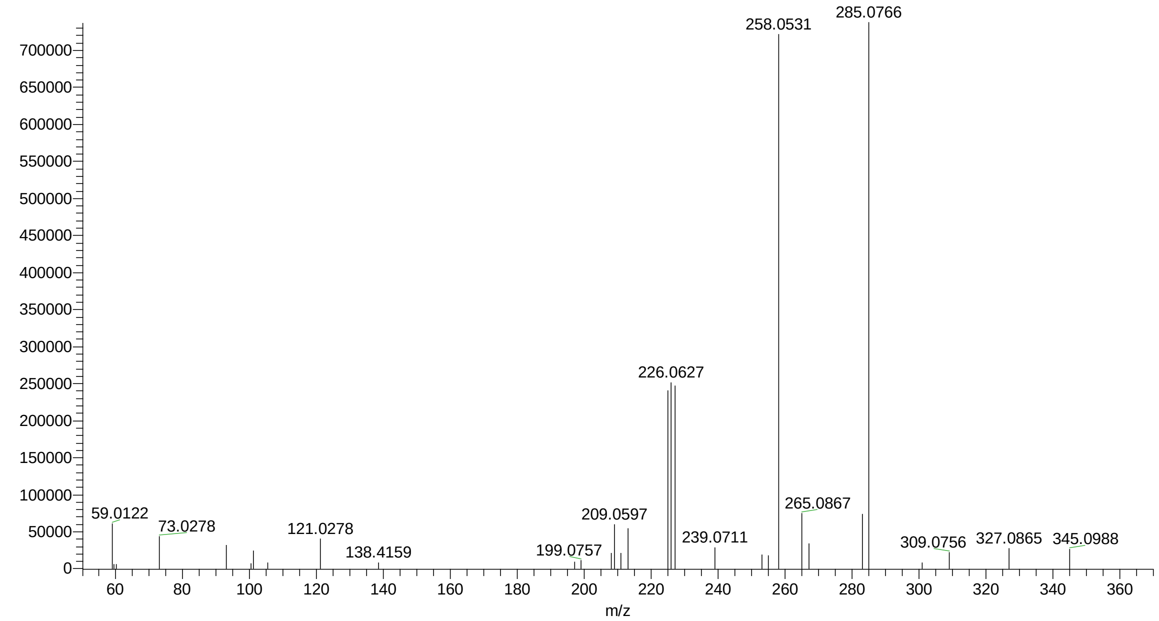

Figure S7. HR-MS/MS spectrum of compound **1b**

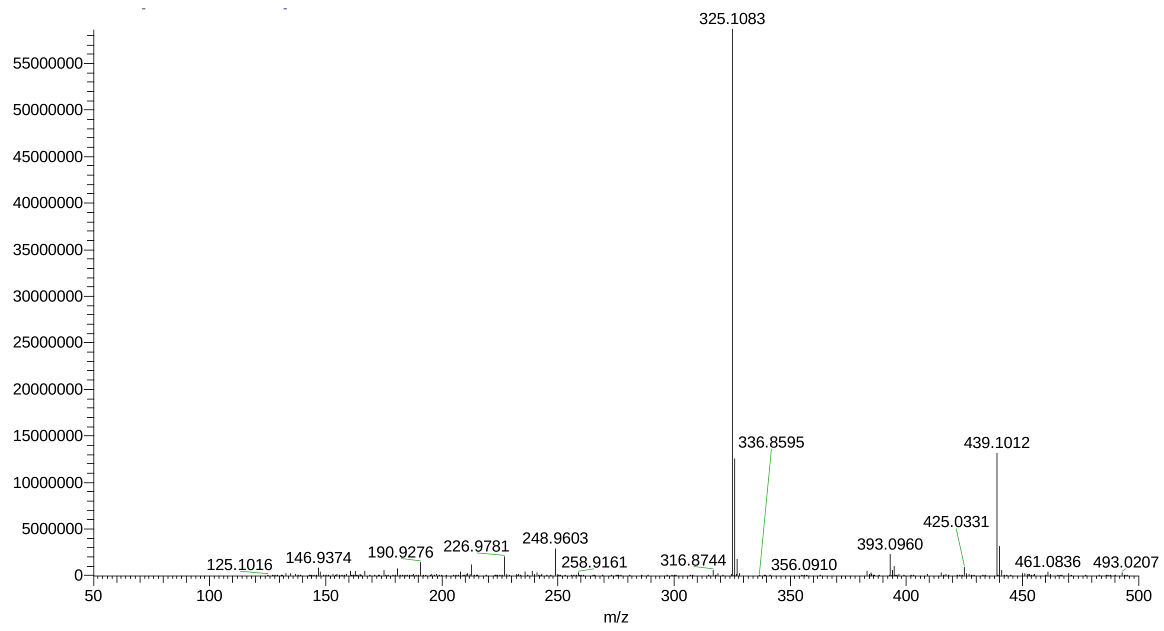

Figure S8. HR-ESI-MS spectrum of compound **1c**

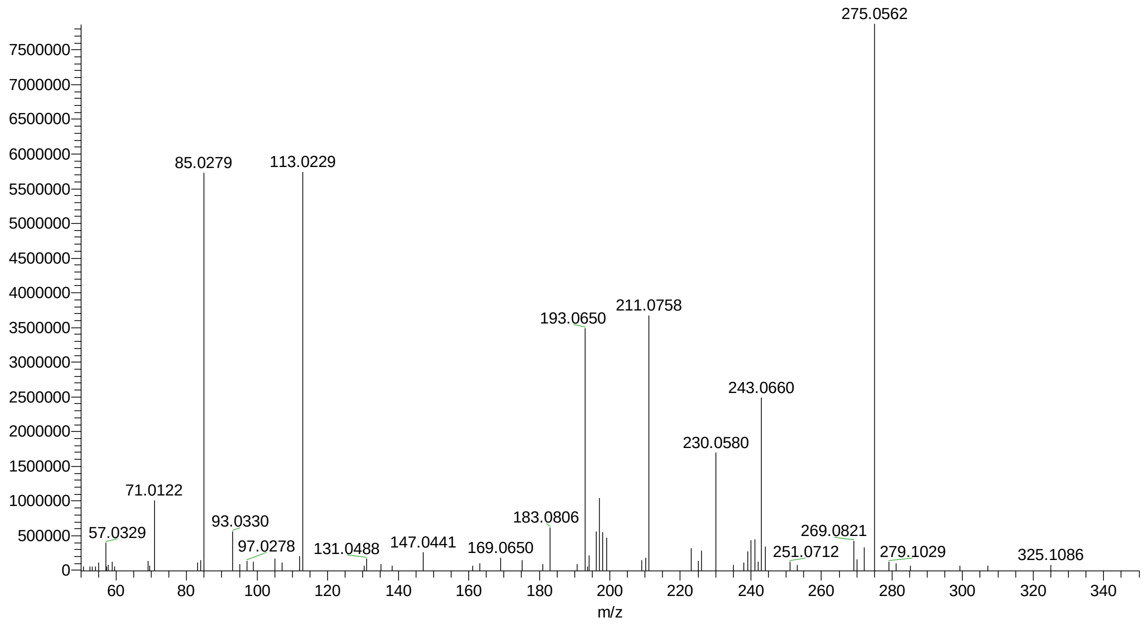

Figure S9. HR-MS/MS spectrum of compound **1c**

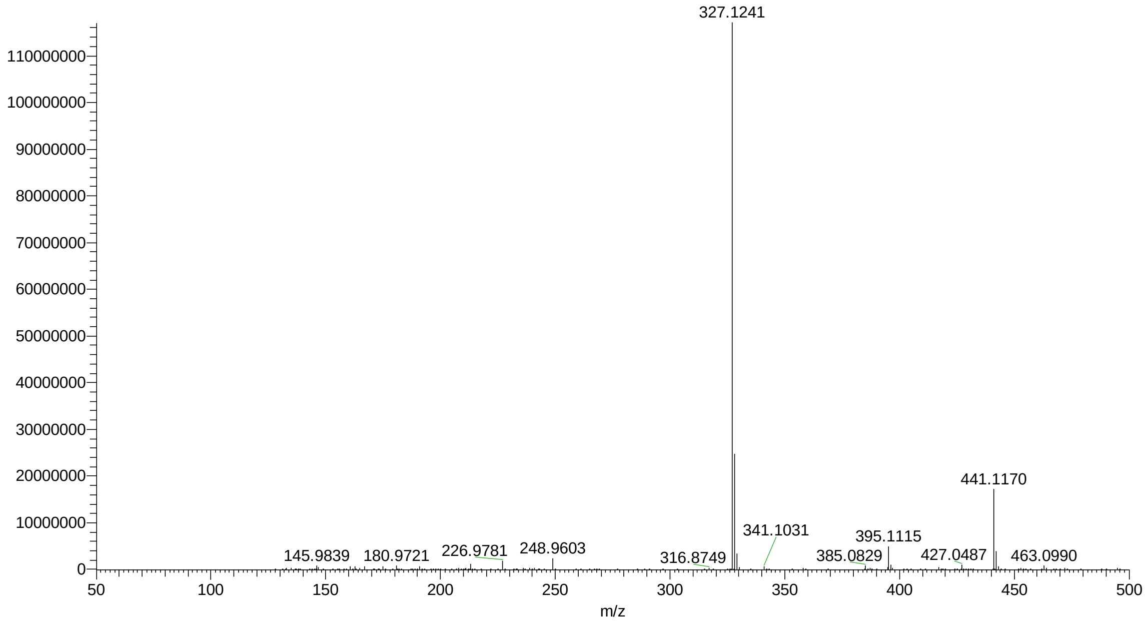

Figure S10. HR-ESI-MS spectrum of compound **3**

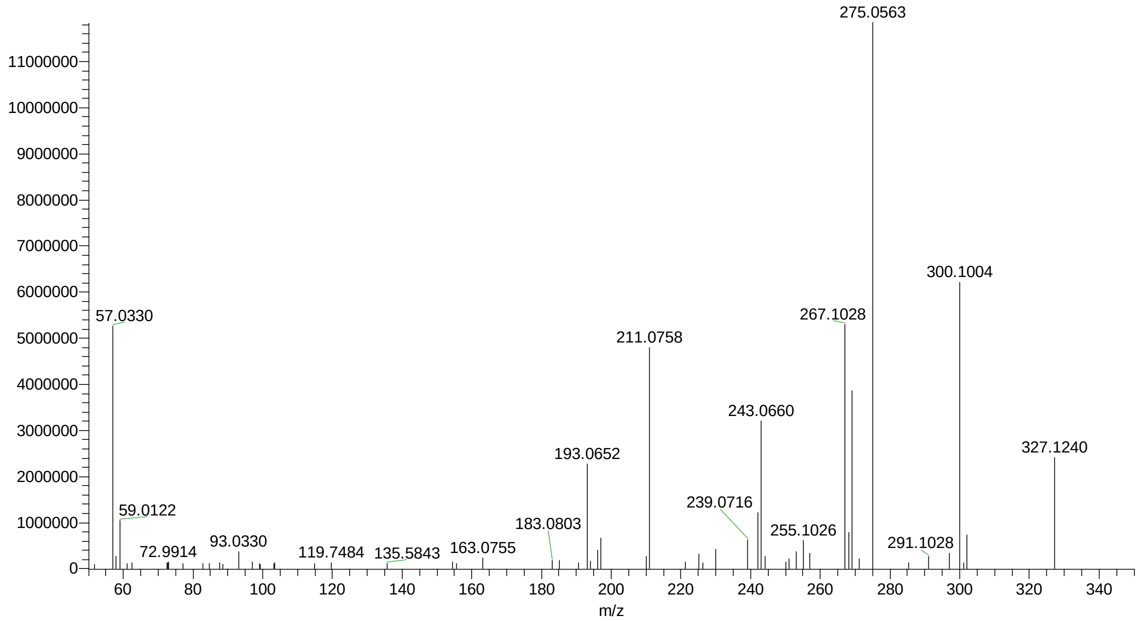

Figure S11. HR-MS/MS spectrum of compound **3**

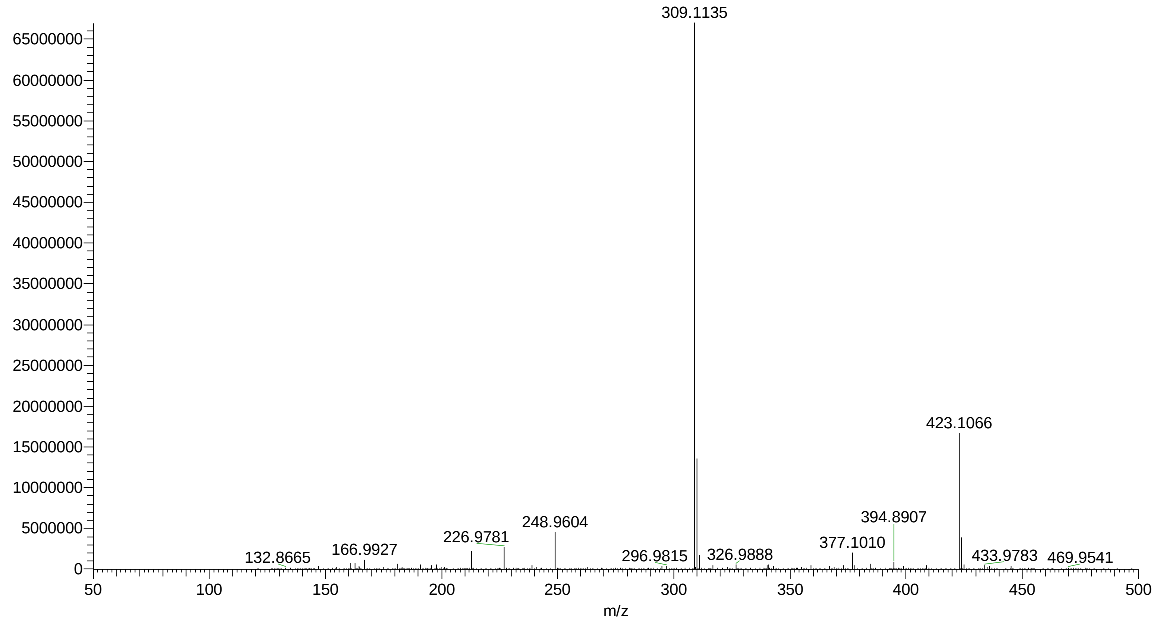

Figure S12. HR-ESI-MS spectrum of compound **3a**

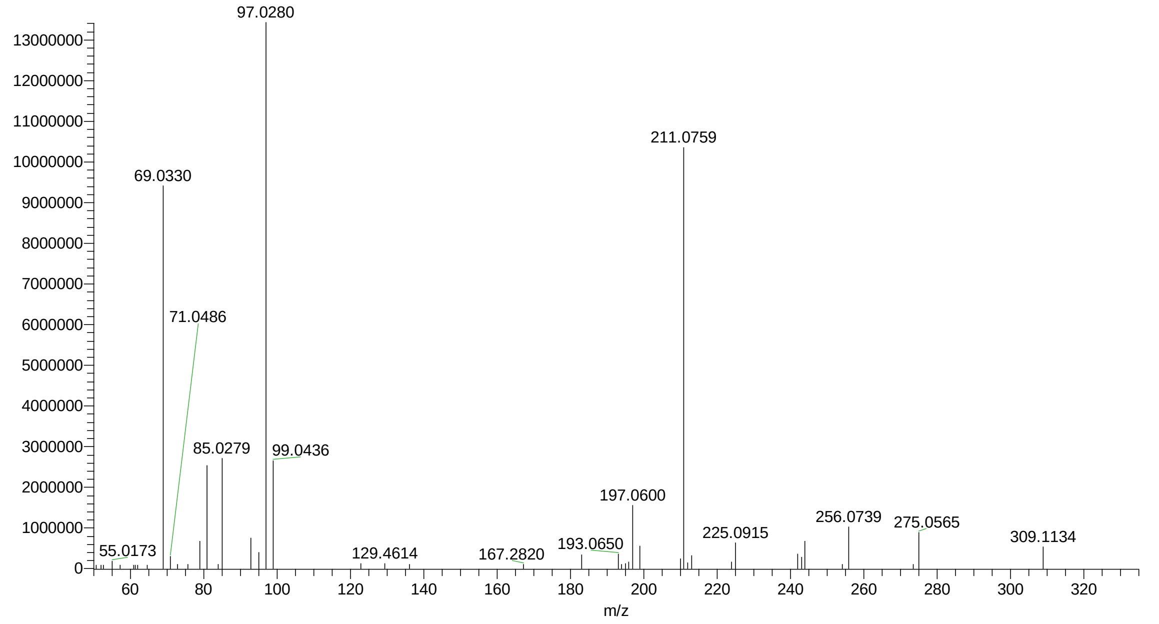

Figure S13. HR-MS/MS spectrum of compound **3a**

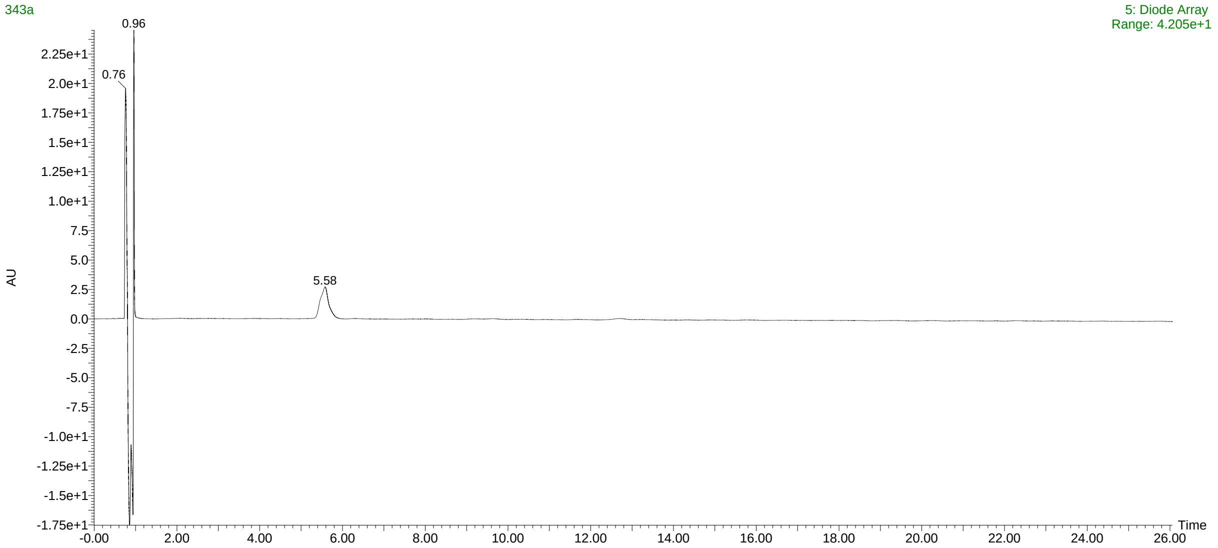

Figure S14. Representative UHPLC-DAD chromatogram of a subfraction containing carpinontriol A (**1**) collected during the final isolation step. For chromatographic conditions see section 3.4.

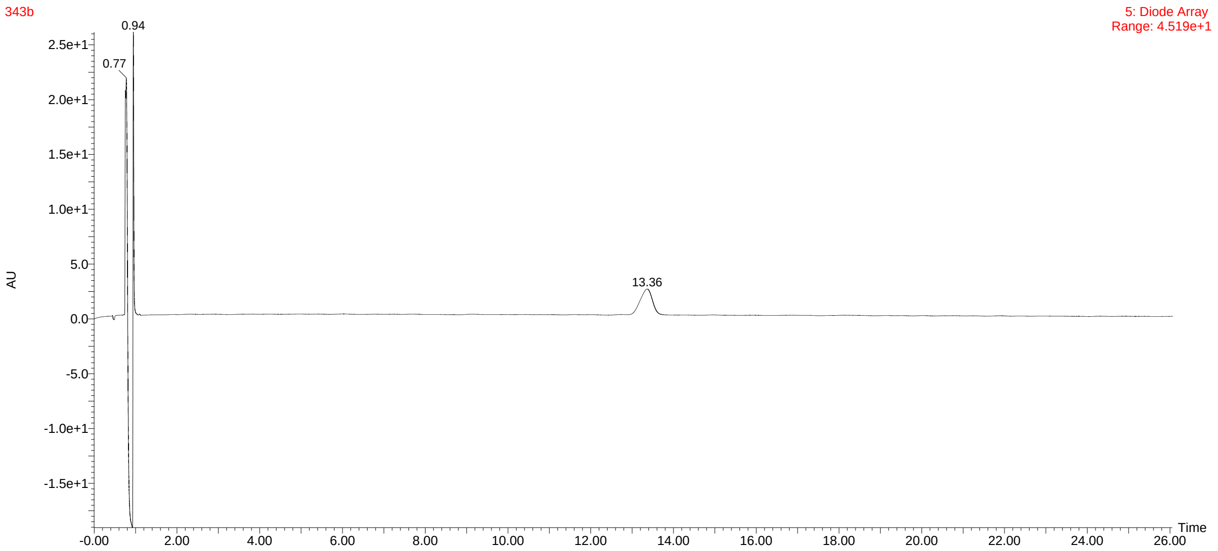

Figure S15. Representative UHPLC-DAD chromatogram of a subfraction containing carpinontriol B (**2**) collected during the final isolation step. For chromatographic conditions see section 3.4.

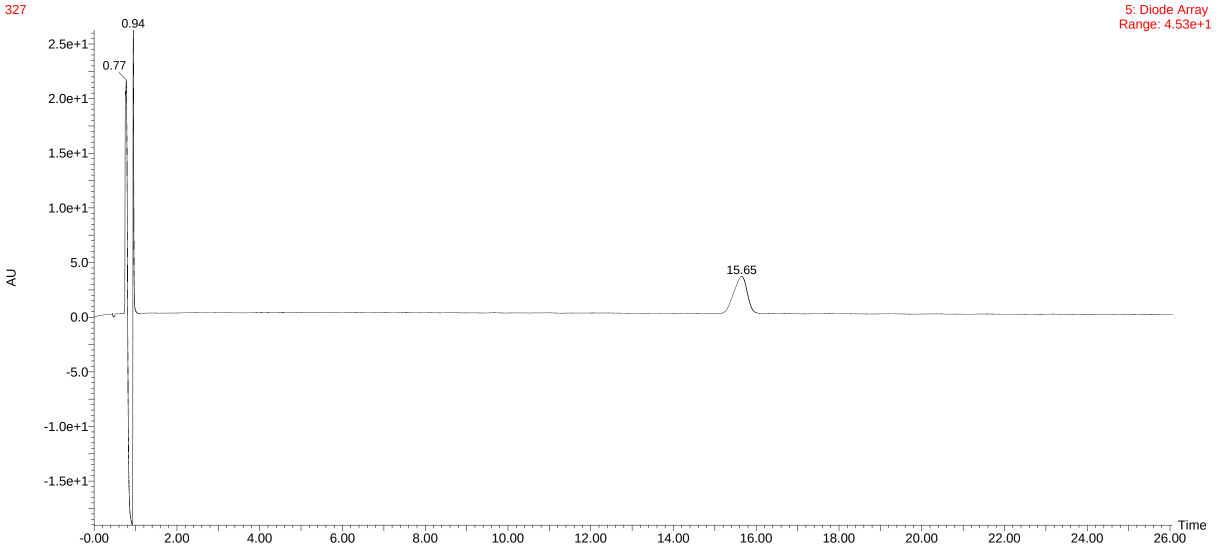

Figure S16. Representative UHPLC-DAD chromatogram of a subfraction containing giffonin X (**3**) collected during the final isolation step. For chromatographic conditions see section 3.4.

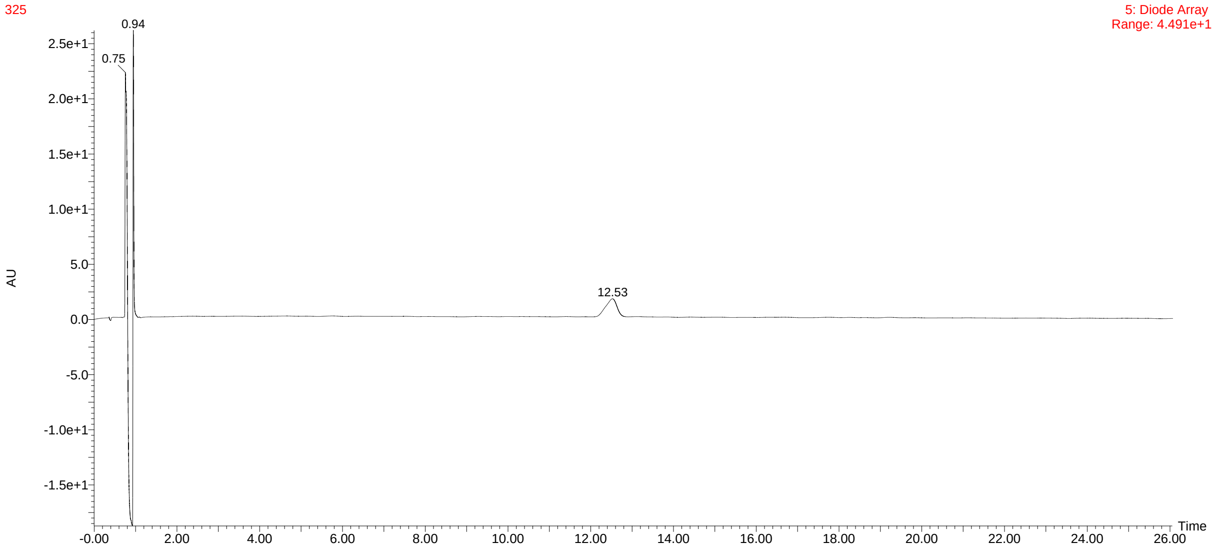

Figure S17. Representative UHPLC-DAD chromatogram of a subfraction containing 3,12,17-trihydroxytricyclo [12.3.1.1^{2,6}]nonadeca-1(18),2(19),3,5,14,16-hexaene-8,11-dione (**4**) collected during the final isolation step. For chromatographic conditions see section 3.4.
